# Effectiveness and Quality of Implementing a Best Practice Model of Care for Low Back Pain (BetterBack) Compared with Routine Care in Physiotherapy: A Hybrid Type 2 Trial

**DOI:** 10.3390/jcm10061230

**Published:** 2021-03-16

**Authors:** Karin Schröder, Birgitta Öberg, Paul Enthoven, Henrik Hedevik, Maria Fors, Allan Abbott

**Affiliations:** 1 Unit of Physiotherapy, Department of Health, Medicine and Caring Sciences, Linköping University, S-581 83 Linköping, Sweden; birgitta.oberg@liu.se (B.Ö.); paul.enthoven@liu.se (P.E.); henrik.hedevik@liu.se (H.H.); maria.fors@liu.se (M.F.); 2 Department of Activity and Health in Linköping, Linköping University, S-581 83 Linköping, Sweden

**Keywords:** low back pain, practice guideline, primary health care, treatment outcome, cluster randomized controlled trial, implementation, rehabilitation, physiotherapy

## Abstract

Low back pain (LBP) occurs in all ages and first-line treatment by physiotherapists is common. The main aim of the current study was to evaluate the effectiveness of implementing a best practice model of care for LBP (intervention group—BetterBack☺ MoC) compared to routine physiotherapy care (control group) regarding longitudinal patient reported outcomes. The BetterBack☺ MoC contains clinical guideline recommendations and support tools to facilitate clinician adherence to guidelines. A secondary exploratory aim was to compare patient outcomes based on the fidelity of fulfilling a clinical practice quality index regarding physiotherapist care. A stepped cluster randomized design nested patients with LBP in the three clusters which were allocated to control (*n* = 203) or intervention (*n* = 264). Patient reported measures were collected at baseline, 3, 6 and 12 months and analyzed with mixed model regression. The primary outcome was between-group changes from baseline to 3 months for pain intensity and disability. Implementation of the BetterBack☺ MoC did not show any between-group differences in the primary outcomes compared with routine care. However, the intervention group showed significantly higher satisfaction at 3 months and clinically meaningful greater improvement in LBP illness perception at 3 months and quality of life at 3 and 6 months but not in patient enablement and global impression of change compared with the control group. Physiotherapists’ care that adhered to all clinical practice quality indices resulted in an improvement of most patient reported outcomes with a clinically meaningful greater improved LBP illness perception at 3 months and quality of life at 3 and 6 months, significantly greater improvement in LBP illness perception, pain and satisfaction at 3 and 6 months and significantly better enablement at all time points as well as better global improvement outcomes at 3 months compared with non-adherent care. This highlights the importance of clinical guideline based primary care for improving patient reported LBP outcomes.

## 1. Introduction

Low back pain (LBP) is a common recurrent condition and one of the most common reasons for consulting primary care [1]. In Sweden and in many other countries, patients with LBP have direct access to primary care and first-line treatment by physiotherapists (PTs) is common [2,3]. Early physiotherapy for LBP has been shown to lower health care utilization and costs [4]. To assist clinicians in providing evidence-based practice (EBP), clinical practice guidelines for LBP have been developed internationally [5,6] including clinical practice guidelines aiming at physiotherapy management of LBP [7].

Current guidelines have placed greater emphasis on information/education, self-management, and recommend active treatments that address psychosocial factors to prevent patients’ pain to become chronic [8]. Guidelines also recommend against use of passive modalities, referral to secondary care and routine use of medical imaging for benign LBP [6]. However, the poor uptake of these guidelines has been identified as an evidence-to-practice gap internationally [9]. Research has focused on investigating the effectiveness of different guideline implementation strategies [10,11,12], but the effects of these implementation strategies on health care practitioners’ behavioral change and patient outcomes are scarce [13,14]. Implementation in the physiotherapy context has been shown to be a challenge [13,15,16]. In a systematic review about implementation interventions for musculoskeletal conditions that included 24 LBP studies, positive changes in PTs’ attitudes, beliefs and skills were reported. However, no consistent improvements in clinical practice and patient outcomes were observed [13]. Another systematic review on guideline implementation in physiotherapy based on only two LBP studies [15,17] showed similar results with no change in patient outcomes [16]. Studies on implementation of LBP guidelines in primary care are even more limited [16,18].

It has been suggested that the implementation of LBP guidelines may improve the quality of health care but there is little research on the impact of PTs’ fulfillment of clinical practice quality indices on patient reported outcomes [19]. This knowledge gap demands more well-designed studies investigating effects of implementation of LBP guidelines in physiotherapy care and especially studies evaluating if PTs’ behavioral change can affect patient outcomes [16]. To facilitate PTs’ guideline adherence, a best practice clinical guideline-based model of care for LBP (BetterBack☺ MoC) has been developed and with a sustained multifaceted strategy implemented in the Swedish primary care setting [20]. This led to improved confidence and biopsychosocial treatment orientation among 116 PTs after implementation of the BetterBack☺ MoC [21]. Furthermore, implementation of the BetterBack☺ MoC also showed a change in the proportions of patients receiving guideline adherent care from 26% before to 59% after the implementation (under review). One can hypothesize that these positive changes in PTs’ confidence, biopsychosocial treatment orientation and guideline adherent behavior may potentially have larger effects on the patient reported outcomes than routine care. Therefore, the primary aim of this study was to investigate if a sustained multifaceted implementation strategy for the BetterBack☺ MoC will result in more statistically significant and greater clinically important difference compared with routine care for LBP. This regarding longitudinal patient-reported measures for LBP intensity, disability, illness beliefs, quality of life, self-care enablement, global rating of change and satisfaction. A secondary exploratory aim was to compare patient outcomes based on the fidelity of clinical practice quality index (CPQI) adherence regarding PTs’ care.

## 2. Materials and Methods

### 2.1. Study Design

This study is a single blinded stepped cluster randomized controlled trial nested within a hybrid type 2 effectiveness-implementation trial [20], hypothesizing superiority of outcomes after implementation of BetterBack☺ MoC compared to previous routine care. The study has followed an a-priori published research protocol [20] prospectively registered in ClinicalTrials.gov Identifier: NCT03147300. An additional exploratory analysis was performed to investigate patient outcomes contingent to the fidelity of CPQI fulfillment regarding PTs’ care. The three clusters were based on the existing geographical and organizational structure to minimize contamination between clusters. During the study time period there were no other organized joint educational activities between clusters.

Random concealed allocation was used where the unit of randomisation was the three primary care rehabilitation unit organizational clusters within the health care region of Östergötland, Sweden. Random concealed allocation was performed by a blinded researcher (AA) randomly selecting three sealed opaque envelopes containing the organizational cluster information. One researcher (KS) informed the clinics in the three clusters of their allocation either to routine care or intervention study condition. The PTs in participating units (practitioner level) and their patients (patient level) were nested within these three clusters. Study participants received routine care or BetterBack☺ MoC dependent upon a stepped cluster [22] dogleg structure [23]. This involved one cluster of PTs immediately receiving education in use of the BetterBack☺ MoC which could influence their management of patients throughout the study (Intervention group). A second cluster of PTs treated patients according to routine care (Control group) in a first phase, and later, after having received education the same PTs treated new patients according to the BetterBack☺ MoC throughout the rest of the study (Intervention group). A third cluster of PTs treated patients according to routine care throughout the trial (Control group) [23]. The PTs and patients in the control group were blinded as they were unaware of content and the difference between routine care and BetterBack☺ MoC. Outcomes were measured at baseline, 3, 6 and 12 months. The trial is reported according to the StaRI checklist for implementation studies [24]. Ethical clearance for the study has been attained through the Regional Ethics Committee in Linköping.

### 2.2. Participants and Setting

All three primary care rehabilitation organizational clusters in Östergötland health care region with a total of 123 PTs were invited to participate in the study. Patients were consecutively recruited by PTs from the clusters after written consent. The inclusion criteria for patients were: 18–65 years, fluent in Swedish and accessing public primary care due to a first-time or recurrent episode of acute, subacute, or chronic-phase benign LBP with or without radiculopathy. Exclusion criteria were: current diagnosis of a malignancy, previous malignancy the last 5 years, infection, spinal fracture, cauda equina syndrome, spinal surgery the last 2 years, current pregnancy or previous pregnancy up to 3 months, ankylosing spondylitis or rheumatic disease, participants who fulfil the criteria for multimodal/multiprofessional rehabilitation for complex long-standing pain and severe psychiatric diagnosis.

### 2.3. Participants and Setting

#### 2.3.1. Control Group

Patients in the control group received routine physiotherapy care at their local PT clinic with no content, dosage, or frequency restriction [20]. PTs delivering routine care had no knowledge of or training in the use of the BetterBack☺ MoC when their regional organizational cluster was in the control group phases of the study.

#### 2.3.2. Intervention Group

The BetterBack☺ MoC was based on international guidelines and was locally adapted to the Swedish context [25,26,27]. To support the development, implementation, and evaluation, the behavior change wheel and an international framework was used [28,29,30,31]. Patients in the intervention group received health care from PTs who had undergone education in the use of the BetterBack☺ MoC. It was hypothesized that PTs would apply the BetterBack☺ MoC in patient care and that this would enable patient’s understanding of LBP, coping with LBP and develop self-management strategies grounded in the Common-Sense Model of Self-Regulation [32]. The following is a description of the PTs´ education and the content of the BetterBack☺ MoC structured according to a template for intervention description and replication (TIDieR) [33]:

- Why: The main PT target behavior was the adoption of the BetterBack☺ MoC to influence delivery of care coherent with best practice clinical guideline recommendations.
- What: This would require the contents of the BetterBack☺ MoC as outlined in Supplementary File S1 to change barrier behaviors such as PTs having low confidence in skills/capabilities for improving LBP patient management and low awareness of evidence based clinical guidelines or coordinated care pathways.
- How: BetterBack☺ MoC content used to overcome the modifiable barriers were support tools including clinical practice guidelines, patient-centered coordinated care pathway, assessment and clinical reasoning tools, patient education brochures and group education material on LBP and self-care, as well as functional restoration program resources.
- When/How much/Tailoring: Intervention delivery, dosing, frequency and progression was stratified based on the PTs’ clinical reasoning regarding risk of pain persistence towards patient’s goals and was delivered at local PT clinics.
- Procedure: A flow diagram for content delivery was provided in the BetterBack☺ MoC. A sustainable multifaceted implementation strategy for PTs use of the BetterBack☺ MoC was composed of the following 3 main facets: (1) Involving an already existing regional implementation steering group including clinic managers who requested an improvement of LBP care and the clinical implementation researchers responsible for overarching logistics; (2) Forming a regional MoC support team comprised of experienced PTs (clinical champions) as local clinic based MoC ambassadors; (3) PT workshops (13.5 h) conducted by the regional support team and steering group at baseline and 3 months (2 h) and a web-based education module for BetterBack☺ MoC users. The behavior change wheel [28] was applied as a theoretical basis for the PT workshops where functions such as education and persuasion about evidence-based recommendations for LBP care as well as training and modelling of the practical use of the BetterBack☺ MoC were used. Detailed information about development and the multifaceted implementation strategy of the BetterBack☺ MoC can be found in the a-priori published protocol [20].

### 2.4. Patient Reported Outcome Measures (PROMs)

#### 2.4.1. Primary Outcome Measures

All patient reported outcomes were collected using paper-based questionnaires at baseline administrated by PTs or the health care clinic reception staff. The paper-based questionnaires at 3, 6 and 12 months follow-up were distributed by mail. In line with the published a-priori study protocol [20], the primary outcomes were mean differences between the control and intervention groups regarding change from baseline to 3 months follow-up for LBP intensity evaluated with Numeric Rating Scale (NRS-LBP) [34] and function and activity limitations using Oswestry Disability Index (ODI) [35]. Both NRS-LBP and ODI have been recommended in international research and both with sound psychometric properties [36,37]. NRS-LBP is a numerical pain intensity rating scale ranging from 0 (no pain) to 10 (worst pain imaginable) [34]. ODI includes ten items related to how LBP affects common daily activities and pain intensity, with six answer options (0–5) for each item, generating a sum score transformed to 0–100% disability (0% no disability due to LBP to 100% completely disable due to LBP) [35].

#### 2.4.2. Secondary Outcome Measures

Secondary outcomes were within group NRS-LBP and ODI longitudinal changes from baseline to 3, 6 and 12 months follow-up and mean differences between the control and intervention groups regarding change from baseline to 6 and 12 months follow-up. Further secondary outcomes were mean change within and between groups from baseline to 3, 6 and 12 months in the following PROMs: health related quality of life assessed by European Quality of life instrument (EQ-5D) ranging from −0.59–1.00 where a higher score indicates better health [38]. Cognitive and emotional representations of illness assessed by Brief Illness Perception Questionnaire (BIPQ) ranging from 0–80 where higher scores indicate more threating views of the illness [39]. Patient´s ability to understand and cope with LBP assessed by Pain Enablement Instrument (PEI) ranging from 0–12 where higher scores indicate better/more enablement [40]. Furthermore, patient reported experience measures were used to evaluate treatment effect assessed by Patient Global Impression of Change (PGIC) [41], and satisfaction with LBP care assessed by Patient Satisfaction (PS) [42]. PGIC was measured on a categorical scale range from −5 (very much worse) thru 0 (unchanged) to 5 (completely recovered) and this was dichotomized into not improved = −5 to 0 and improved = 1 to 5. PS was measured on a categorical scale ranging from very satisfied (1), somewhat satisfied (2), neither satisfied nor dissatisfied (3), somewhat dissatisfied (4), or very dissatisfied (5). The scale was dichotomized into satisfied = 1 and 2, dissatisfied = 3 to 5. PEI, PGIC and PS were not measured at baseline since they are transition rating scales assessed at 3, 6 and 12 months.

In the secondary analysis, clinical practice quality indices regarding PTs’ care were collected from a public health-care regional registry by the researchers as well as from PTs’ reported choice of treatment for each patient in a paper-based clinical reasoning and process evaluation tool (CRPE). PTs were instructed to complete the CRPE tool at the first and last consultations for all patients presenting with a new or recurrent episode or LBP during the study period. The tool is a standardized assessment, that enables analysis of the PTs’ treatment protocol that was based on International Classification of Functioning, Disability and Health brief core set for LBP [43] and health care intervention codes. The CRPE tool (Supplementary File S1) was designed to minimize PTs’ workload with categorical variables and few free text answers. The resulting CPQI contains two assessment and three treatment clinical practice quality indices (Table 1). For PTs’ care considered as fulfilling the CPQI, the delivery of care to patients had to have all five clinical practice quality indices fulfilled and this group is defined as the CPQI adherent care group. The group with less than 5 clinical practice quality indices fulfilled are defined as the non CPQI adherent care group.

### 2.5. Data Analysis

The study cohort specific minimal clinically important difference (MCID) was assessed for primary and secondary outcomes at 3, 6 and 12 months follow-up to interpret the within and between-group changes. MCID was based on recommendations for use of an anchor method where PGIC could serve as a sufficiently strong anchor when correlation coefficients are ≥0.30 in association to PROMs [44]. To interpret MCID, the optimal cut-off point (OCP) was used. The OCP on a Receiver Operating Curve (ROC) was defined by the sensitivity and specificity with lowest percentage of misclassified regarding improvement on the PGIC [45]. As a secondary criterion, if the OCP indicated worsening of the PROM, the nearest value indicating an improvement on the PROM was chosen as the MCID. Area Under the Curve (AUC) ≥ 0.70 represents satisfactory accuracy for the model [46,47]. 

Data collected at different time points were analyzed according to an intention to treat principle. This by using restricted maximum likelihood approach in mixed models adjusted for unstructured covariance structure [48]. Linear mixed models were used for continuous dependent variables and generalized mixed models were used for dichotomous dependent variables. Intraclass correlation coefficients were calculated for clustering effects of the three regions that formed the control and intervention groups [23]. The cluster variable was entered into the mixed models as a random effect, while a longitudinal time variable as well as the control/intervention grouping variable or CPQI variable were entered as fixed effects. The primary endpoint analysis for primary outcomes variables involved testing a control/intervention between group-by-time interaction specifically for the pairwise contrast for change from baseline to 3 months with a significance level of *p* = 0.05 in line with the a-priori protocol. Secondary endpoint between group pairwise contrasts were also analyzed for the time points baseline to 6 months and baseline to 12 months. Furthermore, secondary analysis of within group changes from baseline across all follow-up assessments were analyzed for all outcome measures with a Bonferroni adjusted two-sided significance level of *p* ≤ 0.017. All statistical analyses were performed using IBM SPSS version 25 and with the statistical package R 3.6.0 (R Core Team, 2018). Based on a-prior hypothesized small effect size (*d* = 0.35) on changes in patients’ primary and secondary outcomes and with a one tailed *p* = 0.05 for the superiority of the intervention compared with the control group with 80% statistical power 204 participants were needed. Adjusting for the design effect due to cluster randomization, an intra-cluster correlation of 0.01, a cluster autocorrelation of 0.80, a dog leg stepped design with 100 participants in each cluster, a total of 402 participants over 2.41 clusters were needed for 80% statistical power [23]. To overcompensate for a potential unbalanced recruitment flow and increasing dropout in the longitudinal outcomes, the original target was to recruit up to 600 participants between April 2017 to January 2018.

## 3. Results

### 3.1. Participant Flow and Baseline Characteristics

**Figure 1:**
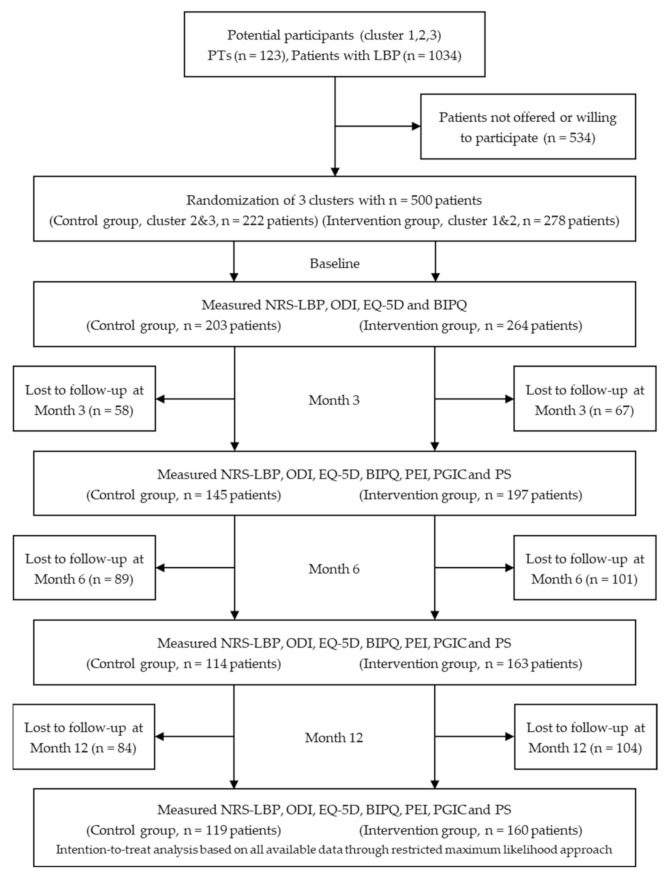
Flow diagram of participants throughout the trial. NRS-LBP = Numeric Rating Scale-Low Back Pain, ODI = Oswestry Disability Index, EQ-5D = EuroQol 5 dimensions, BIPQ = Brief Illness Perception Questionnaire, PEI = Patient Enablement Instrument, PGIC = Patient Global Impression of Change, PS = Patient Satisfaction.

From a total of 1034 consecutive patients with LBP seeking physiotherapy in public financed primary care in the region of Östergötland between April 2017 to January 2018, 500 fulfilled inclusion and exclusion criteria as well as accepted participation in the study (Figure 1). Cluster randomization allocated 222 patients to the control group and 278 to the intervention group. Baseline PROMs were attained for 467 of these patients, 203 in the control group, and 264 in the intervention group. Furthermore, data forming the CPQI was attained for 355 patients with 164 patients receiving physiotherapy care with CPQI adherence and 191 patients receiving physiotherapy care that was non CPQI adherent.

Baseline demographics and baseline clinical characteristics of the study participants were similar in the intervention and control groups and are presented in Table 2. PTs who treated patients in the control and intervention groups were similar in gender, age, clinical experience, and education level. There were also no statistically significant differences in patient characteristics between responders to longitudinal follow-ups and non-responders. Furthermore, there were no statistically significant differences in characteristics between the 112 patients (24%) without and the 355 patients with PTs reported data forming the CPQI.

### 3.2. Minimum Clinically Important Differences in PROMs

MCID for change in primary and secondary outcomes at 3, 6 and 12 months are displayed in Table 3. MCID values for PROMs at each time point sufficiently correlated with PGIC and attained a satisfactory AUC accuracy level except for EQ-5D at 6 months for which it’s MCID should be interpreted with caution.

### 3.3. Patient Outcomes Based on Control and Intervention Group within and Between-Group Effects 

As outlined in Table 4 and Table 5 There were no statistically significant between-group differences in the primary or secondary outcomes and endpoints except for a higher proportion of patients reporting satisfaction with the LBP care after 3 months in the intervention group compared with the control group (OR 1.7, 95% CI 1.5–1.9, *p* < 0.001). There was a clinically meaningful larger between-group improvement in the EQ-5D from baseline to 3 and 6 months and BIPQ from baseline to 3 months to the advantage for the intervention group. 

The within-group analyses for ODI, NRS-LBP, EQ-5D and BIPQ in both the control and intervention group showed a statistically significant (*p* < 0.001) and clinically meaningful improvement from baseline to 3, 6 and 12 months follow-ups except for no clinically meaningful improvement in NRS-LBP at 6 months in the control group and in BIPQ at 12 months in both groups. PEI had clinically meaningful improvement on transition scores at each time in both the control and intervention groups.

### 3.4. Patient Outcomes Based on the Fidelity of CPQI Adherence Regarding PTs’ Care

As outlined in Table 6, the between group analysis showed that the CPQI adherent care group showed a statistically significant larger improvement in PEI throughout all follow-ups as well as BIPQ and NRS-LBP from baseline to 3 months and 6 months. There was also a clinically meaningful larger between-group improvement in the BIPQ from baseline to 3 months and in EQ-5D from baseline to 3 and 6 months to the advantage for the CPQI adherent care group. The within-group analyses for both groups showed statistically significant (*p* < 0.001) and clinically meaningful improvement from baseline to 3, 6 and 12 months follow-ups in ODI, NRS-LBP, EQ-5D and BIPQ except for NRS-LBP at 6 months and BIPQ at 6 and 12 months in both groups. 

Table 7 presents patient reported experience measures showing a statistically significant higher proportion of patients reporting satisfaction with the LBP care after 3 months (OR 2.2, 95% CI 1.3–4.0, *p* = 0.006) and 6 months (OR 2.7, 95% CI 1.5–6.4, *p* = 0.001) in the CPQI adherent care group compared with the non CPQI adherent care group. PGIC was also statistically significant higher after 3 months (OR 2.3, 95% CI 1.2–4.2, *p* = 0.009) in the CPQI adherent care group compared with the non CPQI adherent care group.

## 4. Discussion

This study is among the first to evaluate what effect implementation of a best practice clinical guideline based MoC for LBP compared to routine physiotherapy care has on patient outcomes and evaluate fidelity of clinical practice quality on patient outcomes within the same study. Implementation of the BetterBack☺ MoC compared to routine physiotherapy did not provide better primary and secondary outcomes except higher patient satisfaction with LBP care at 3 months as well as short-term clinically meaningful improvements in quality of life and LBP illness perception. A higher satisfaction in the intervention group could be explained by the slightly larger number of treatment sessions in the intervention group (3.8) compared to control group (2.7). However, this larger number can be explained by the more often use of group training intervention as part of the BetterBack☺ MoC that yields a higher number of treatment sessions compared to the more often used home-based exercise in the control group. Other potential reasons for higher satisfaction in the intervention group have been shown in a qualitative study where patients in the BetterBack☺ MoC group experienced better knowledge about their LBP and received tools to better manage their health condition [49]. Patient satisfaction with care has been seen to correlate with quality of life, but the directionality needs further investigation [50]. Bamm et al. [50] suggest that although change in the way we organize and provide treatment might not lead to significant changes in patients’ function and activity, clinicians can potentially affect patient’s perception of LBP and improve their coping with illness. Difficulties in showing between-group differences in PROMs is in line with the few existing studies evaluating patient outcomes after implementation of LBP guidelines [18,51]. A recent systematic review by Bérubé et al. [13] found only eight studies evaluating patient outcomes after various implementation strategies in LBP physiotherapy contexts. Only five of these studies were considered of good quality and among these five, only the study by Benecuik and George [52] could show improved patient outcomes after a single faceted implementation of a stratified primary care model. This study by Benecuik and George [52] involved 109 participants with LBP but was later replicated with a larger patient cohort of 1701 patients by Cherkin et al. [53] with no differences in patient outcomes resulting between intervention and control groups.

Guideline implementation with many components is more complex and challenging compared to implementation of a single-component intervention [54,55]. Focusing on LBP guideline implementation, only four of the studies included in the systematic review by Bérubé et al. [13] evaluated the effects of LBP guideline implementation in physiotherapy settings on patient outcomes with no change in quality of life [56], function and disability [15,57] and pain intensity [15]. There can be multiple reasons why implementation of complex interventions had difficulties in improving patient outcomes compared to various control groups. Low quality and quantity of the implementation strategies aimed to change practice [16] and too short follow-ups after implementation for practitioners’ behavioral change to occur has been suggested [13]. Rapid and substantial improvements as seen also in the current study is often limited to short term effects in LBP studies [58]. Furthermore, only small differences in clinical guideline adherence between intervention and control groups has been reported in previous research, ranging between 5–12% [59,60]. However, in the current study cohort, we have previously reported that CPQI adherence was already existent in 26% of PTs’ care delivery for the control group and despite significant improvement by 35% after implementing the BetterBack☺ MoC, there were still 41% of PTs with non CPQI adherent care (unpublished data). On a group level, this potentially dilutes the effects of improved PTs’ clinical practice quality to be able to significantly improve patient outcomes. Therefore, exploring patient outcomes based on the fidelity of CPQI adherence regarding PTs´ care is motivated.

The secondary explorative analysis in this study showed that the fidelity of CPQI adherence regarding PTs’ care significantly improved most patient outcomes compared to non CPQI adherent care. Fritz et al. [61] has in a retrospective case control design found that adherence to evidence-based guidelines was associated with 11.3% higher improvement in pain and 16.2% higher improvement in disability over 3 months compared to non-adherence. The current study showed similar improvement in pain but lower improvement in disability due to lower baseline values. The current study has also a stronger design and additional patient outcomes measuring LBP illness perception, patient enablement, satisfaction, and global impression of change. When interpreting the results in the light of the study specific MCID, smaller changes are of importance in our patient group than previously reported MCID for ODI and NRS for other populations [62]. Another interesting result in the secondary exploratory analysis is the between group differences over time regarding pain, LBP illness perception, patient enablement, satisfaction, and global improvement in favor of patients revieing CPQI adherent care. However, these differences were over the cut off for study specific MCIDs for only LBP illness perception in the short term. According to the Common-Sense Model of Self-Regulation [32], providing patients with adequate understanding of illness and its management, may increase motivation and adherence to treatment [32]. Since LBP fluctuates over time and relapse is common, patients’ improved use of self-management strategies that sustain over time are of importance to manage flare-ups and reduce chronicity, health care utilization and costs. Improvement of patient satisfaction with LBP care supports earlier findings where high satisfaction has been shown to correlate with both greater global improvement as well as pain and disability outcomes [63]. A considerable number of patients with acute to subacute LBP develop chronic LBP [64,65] and to be able to hinder this transition, interventions in the early stage of the condition is important [66]. A randomized trial by Fritz et al. [67] that compared early physiotherapy with usual care showed that early physiotherapy provided faster reduction in disability, fear avoidance and pain-catastrophizing although the PT protocol did not include explicit intervention to address psychosocial factors. This highlights the important of early LBP physiotherapy and further research is needed on how to minimize the transition to chronic LBP.

This study has several strengths including a strong randomized design with large practitioner and participant samples. Different practice sizes from 1–4 PTs up to large practices with more than 20 PTs were included. This mix with smaller and larger practices in both rural and urban areas was similar in the intervention and control groups and also regarding CPQI adherence/non-adherence grouping. The physiotherapists that treated patients in the intervention and control group did not differ in educational level, age or years of clinical experience. The non-response analysis showed no selection bias. Participants’ characteristics were similar to those reported in other primary care settings [68] and an earlier study within the same primary care setting [69] as well as the non-response analysis showing no selection bias. The results are therefore generalizable for different practice sizes and Region Östergötland has educational, income and health levels that are comparable with the whole country [70]. This makes our findings generalizable to Swedish physiotherapy care in primary health care and to similar health care systems internationally. However, generalizability must be interpreted with caution since implementation success depends on a high degree of contextual factors [71].

The use of study specific MCID EQ-5D at 6 months follow up had its weakness where it did not reach the predefined correlation of ≥0.3 with the PGIC and model accuracy AUC ≥ 0.7 so its clinically meaningful improvement should therefore be interpreted with caution. A limitation in the exploratory analysis is that it was not planned a-priori and involved only approximately 70% of patients. A further limitation is that PTs may not fully influence general practitioners’ referral to specialist care or use of medical imaging which can affect PTs’ ability to attain full CPQI adherence. A further development of the current BetterBack☺ MoC is needed towards a multi-professional MoC with involvement of especially general practitioners to improve the health care pathway for this patient group. Validation studies in other contexts are also needed of similar effectiveness-implementation design including outcome measures to evaluate illness perception, enablement, satisfaction with care and global impression of change in addition to traditional core outcomes and clinical practice quality processes. To evaluate evidence based clinical guideline uptake among PTs, a longer phase after implementation may be needed since behavior change is expected to take time.

## 5. Conclusions

Implementation of the BetterBack☺ MoC did not show any between-group differences in pain intensity and disability compared with routine care. However, the secondary outcomes showed a higher patient satisfaction with care and larger clinically meaningful improvement in quality of life and LBP illness perception, but not in patient enablement and global impression of change compared with routine care. When PTs’ care was adherent to clinical practice quality indices, most PROMs improved compared to non-adherent care. This is among the first studies indicating that implementation of PTs’ clinical practice of high quality can improve outcomes among patients with LBP and highlights the importance of addressing enablement and LBP illness perception when evaluating LBP interventions with an added value to traditional outcome measures. Since LBP fluctuates over time and relapse is common, improved patient self-management strategies that sustain over time are of importance to manage future flare-ups.

## Figures and Tables

**Table 1:** Clinical practice quality index for PTs’ care delivery to patients with LBP.

Clinical Practice Quality Indices Forming the Clinical Practice Quality Index	
Assessment quality index	1. No referral to specialist consultation (pain clinic, orthopedic or neurosurgical care) during the physiotherapy treatment period
	2. No imaging during the physiotherapy treatment period
Treatment quality index	1. Use of patient education interventions
	2. Use of exercise interventions
	3. No use of non-evidence-based interventions
Clinical practice quality index	All 5 quality indices fulfilled

**Table 2:** Baseline characteristics of the included patients.

	Control Group (*n* = 203)	Intervention Group (*n* = 264)
Age, mean ± SD	46 ± 12	45 ± 12
Sex, female, *n* (%)	109 (54)	152 (58)
Educational level, *n* (%)		
Elementary	24 (12)	34 (13)
High school	112 (55)	158 (60)
University	66 (33)	71 (27)
Pain Duration, *n* (%)		
<12 weeks	111 (57)	138 (55)
>12 weeks	83 (43)	115 (46)
Employed, *n* (%)	164 (81)	217 (82)
Sick leave due to back pain, *n* (%)	34 (18)	48 (19)
STB risk groups, *n* (%)		
Low risk group	75 (37)	97 (37)
Medium risk group High risk group	102 (50) 26 (13)	132 (50) 35 (13)
Number of PT treatment sessions, mean ± SD, *n*	3.1 ± 2.7, *n* = 165	4.6 ± 3.8, *n* = 223
Duration PT intervention period, mean days ± SD, *n*	59 ± 84, *n* = 164	63 ± 61, *n* = 218

*n* = number of participants, SD = standard deviation, STB = STarT Back Tool.

**Table 3:** Minimal clinically important differences (MCID) interpreted with optimal cut-off point (OCP) at baseline to follow-up for the total study cohort.

PROMs	Correlation with PGIC	Change from Baseline, Mean ± SD	MCID, OCP	Youden Index	(Sensitivity; Specificity)	AUC
**3 months**						
ODI (*n* = 337)	0.45	8.7 ± 15.1	4.5	0.52	(0.69; 0.83)	0.81
NRS-LPB (*n* = 337)	0.39	2.7 ± 2.9	2.5	0.41	(0.58; 0.82)	0.76
EQ-5D (*n* = 320)	0.36	0.12 ± 0.32	0.02	0.39	(0.66; 0.73)	0.74
BIPQ (*n* = 339)	0.52	8.6 ± 16.1	0.5 ^†^	0.56	(0.81; 0.75)	0.87
PEI * (*n* = 335)	0.50	4.4 ± 4.0	2.5	0.59	(0.76; 0.83)	0.86
**6 months**						
ODI (*n* = 270)	0.40	10.4 ± 16.6	4.5	0.44	(0.68; 0.76)	0.78
NRS-LPB (*n* = 270)	0.30	2.6 ± 2.9	2.5	0.35	(0.59; 0.76)	0.70
EQ-5D (*n* = 259)	0.21	0.18 ± 0.31	0.03	0.27	(0.71; 0.56)	0.67
BIPQ (*n* = 273)	0.37	9.4 ± 16.0	8.5	0.40	(0.58; 0.82)	0.74
PEI * (*n* = 265)	0.48	4.6 ± 4.2	3.5	0.55	(0.67; 0.88)	0.82
**12 months**						
ODI (*n* = 270)	0.35	11.9 ± 15.6	8.5	0.38	(0.59; 0.79)	0.75
NRS-LPB (*n* = 273)	0.32	2.9 ± 2.8	1.5	0.35	(0.77; 0.58)	0.72
EQ-5D (*n* = 264)	0.31	0.18 ± 0.32	0.18	0.30	(0.49; 0.81)	0.70
BIPQ (*n* = 271)	0.47	10.8 ± 16.0	12.5	0.49	(0.53; 0.98)	0.82
PEI * (*n* = 261)	0.47	4.9 ± 4.2	2.5	0.53	(0.76; 0.78)	0.82

PROM = Patient Reported Outcome Measure, PGIC = Patient Global Impression of Change, SD = standard deviation, MCID = minimal clinical important difference, OCP = optimal cut-off point, AUC = area under the curve, ODI = Oswestry Disability Index, NRS-LBP = Numeric Rating Scale-Low Back Pain, EQ-5D = EuroQol 5 dimensions, BIPQ = Brief Illness Perception Questionnaire, PEI * = Patient Enablement Instrument, PEI is a transition rating scale with only 3, 6 and 12 months values. † = OCP (−0.5) nearest value indicating improvement on the PROM was chosen as the MCID.

**Table 4:** Comparisons of patient reported outcome measures in control and intervention group.

	Within-Group Analysis of Change from Baseline		Between-Group Effects (1–2) at Each Endpoint	
	1. Control Group (*n* = 203)	2. Intervention Group (*n* = 264)		
	Mean (95% CI) *p*-Value	Mean (95% CI) *p*-Value	Mean (95% CI) *p*-Value	ICC
ODI (0–100)	31.6 (27.2 to 36.1) *	30.4 (25.6 to 35.3) *		
3 months	−10.5 (−13.4 to −7.6) ***p* < 0.001**	−8.7 (−11.2 to −6.2) ***p* < 0.001**	−1.8 (−5.0 to 1.3) *p* = 0.248	0.012
6 months	−10.9 (−14.1 to −7.7) ***p* < 0.001**	−10.2 (−12.9 to −7.5) ***p* < 0.001**	−0.7 (−4.2 to 2.7) *p* = 0.674	
12 months	−14.2 (−17.3 to −11.1) ***p* < 0.001**	−11.3 (−13.9 to −8.6) ***p* < 0.001**	−3.0 (−6.3 to 0.4) *p* = 0.081	
NRS-LBP (0–10)	6.1 (5.6 to 6.7) *	6.4 (5.7 to 7.0) *		
3 months	−2.6 (−3.1 to −2.1) ***p* < 0.001**	−2.9 (−3.4 to −2.5) ***p* < 0.001**	−0.3 (−0.3 to 0.9) *p* = 0.263	0.008
6 months	−2.4 (−3.0 to −1.8) ***p* < 0.001**	−2.7 (−3.2 to −2.2) ***p* < 0.001**	−0.3 (−0.3 to 0.9) *p* = 0.357	
12 months	−3.1 (−3.7 to −2.5) ***p* < 0.001**	−2.8 (−3.3 to −2.3) ***p* < 0.001**	−0.3 (−0.9 to 0.3) *p* = 0.297	
EQ-5D index (−0.59–1)	0.55 (0.50 to 0.60) *	0.52 (0.46 to 0.58) *		
3 months	0.12 (0.06 to 0.18) ***p* < 0.001**	0.15 (0.10 to 0.21) ***p* < 0.001**	−0.03 (−0.10 to 0.04) *p* = 0.381	0.004
6 months	0.13 (0.07 to 0.19) ***p* < 0.001**	0.20 (0.15 to 0.25) ***p* < 0.001**	−0.07 (−0.14 to −0.01) *p* = 0.034	
12 months	0.19 (0.13 to 0.25) ***p* < 0.001**	0.20 (0.14 to 0.25) ***p* < 0.001**	−0.01 (−0.07 to 0.06) *p* = 0.838	
BIPQ total score (0–80)	44.6 (40.4 to 48.8) *	45.4 (40.7 to 50.2) *		
3 months	−8.2 (−11.4 to −5.1) ***p* < 0.001**	−9.0 (−11.6 to −6.3) ***p* < 0.001**	−0.8 (−2.6 to 4.1) *p* = 0.659	0.007
6 months	−9.1 (−12.5 to −5.8) ***p* < 0.001**	−8.8 (−11.6 to −6.0) ***p* < 0.001**	−0.2 (−3.9 to 3.2) *p* = 0.853	
12 months	−11.7 (−15.0 to −8.4)***p* < 0.001**	−10.4 (−13.2 to −7.5) ***p* < 0.001**	−1.3 (−4.9 to 2.2) *p* = 0.457	
PEI (0–12)	Transition score, mean ± SE	Transition score, mean ± SE		
3 months	4.4 ± 0.3	4.5 ± 0.3	−0.1 (−1.0 to 0.7) *p* = 0.768	< 0.001
6 months	4.2 ± 0.4	4.8 ± 0.3	−0.6 (−0.4 to 1.6) *p* = 0.257	
12 months	5.1 ± 0.4	4.9 ± 0.3	0.2 (−1.2 to 0.9) *p* = 0.742	

*n* = number of participants, SE = standard error, CI = confidence interval, ICC = intra-cluster correlation, ODI = Oswestry Disability Index, NRS-LBP = Numeric Rating Scale-Low Back Pain, EQ-5D = EuroQol 5 dimensions, BIPQ = Brief Illness Perception Questionnaire, PEI = Patient Enablement Instrument, PEI is a transition rating scale with only 3, 6 and 12 months values. Bonferroni corrected significance thresholds of *p* ≤ 0.017 are printed in bold. * Mean (95% CI) at baseline.

**Table 5:** Comparisons of patient reported experience measures in control and intervention group.

	1. Control Group, *n*/N (%)	2. Intervention Group, *n*/N (%)	Between-Group Comparison (2/1), OR (95% CI), *p*-Value, ICC
**Patient satisfaction**			
Satisfied after 3 months	93/144 (64.6)	149/197 (75.6)	1.7 (1.5 to 1.9), ***p* < 0.001**, ICC = 0.006
Satisfied after 6 months	68/111 (61.3)	109/163 (66.9)	1.3 (1.0 to 1.6), *p* = 0.027, ICC = 0.002
Satisfied after 12 months	73/116 (62.9)	98/158 (62.0)	0.9 (0.5 to 1.6), *p* = 0.748, ICC < 0.001
**Patient global rating of change**			
Improved after 3 months	105/144 (72.9)	149/197 (75.6)	1.2 (0.7 to 1.9), *p* = 0.570, ICC < 0.001
Improved in after 6 months	74/111 (66.7)	126/163 (77.3)	1.7 (1.0 to 2.9), *p* = 0.054, ICC < 0.001
Improvement after 12 months	86/118 (72.9)	121/158 (76.6)	1.6 (0.7 to 3.9), *p* = 0.290, ICC = 0.035

*n* = number of participants with favorable outcome, N = total number of participants, CI = confidence interval, OR = odds ratio, ICC = intra-cluster correlation, LBP = low back pain. Bonferroni corrected significance thresholds of *p* ≤ 0.017 are printed in bold.

**Table 6:** Comparisons of patient reported outcome measures for patients receiving CPQI adherent/non adherent care.

	Within-Group Analysis of Change from Baseline		Between-Group Effects (1–2) at Each Endpoint	
	1. Non CPQI Adherent Care Group (*n* = 191)	2. CPQI Adherent Care Group (*n* = 164)		
	Mean (95% CI) *p*-Value	Mean (95% CI) *p*-Value	Mean (95% CI) *p*-Value	ICC
ODI (0–100)	32.4 (27.5 to 37.3) *	28.3 (23.5 to 33.2) *		
3 months	−9.0 (−11.8 to −6.2) ***p* < 0.001**	−11.3 (−14.2 to −8.3) ***p* < 0.001**	2.3 (−1.1 to 5.6) *p* = 0.178	0.012
6 months	−8.9 (−12.1 to −6.0) ***p* < 0.001**	−12.7 (−16.1 to −9.4) ***p* < 0.001**	3.8 (0.3 to 7.6) *p* = 0.048	
12 months	−10.7 (−13.9 to −7.6) ***p* < 0.001**	−13.2 (−16.5 to −9.8) ***p* < 0.001**	2.4 (−1.4 to 6.2) *p* = 0.207	
NRS-LBP (0–10)	6.3 (5.5 to 7.1) *	6.1 (5.4 to 6.9) *		
3 months	−2.5 (−3.0 to −2.0) ***p* < 0.001**	−3.4 (−4.0 to −2.8) ***p* < 0.001**	0.9 (0.3 to 1.6) ***p* = 0.004**	0.008
6 months	−2.1 (−2.7 to −1.5) ***p* < 0.001**	−3.2 (−3.8 to −2.6) ***p* < 0.001**	1.1 (0.4 to 1.8) ***p* = 0.002**	
12 months	−2.6 (−3.2 to −2.0) ***p* < 0.001**	−3.1 (−3.7 to −2.5) ***p* < 0.001**	0.5 (−0.2 to 1.2) *p* = 0.169	
EQ-5D index (−0.59–1)	0.51 (0.45 to 0.57) *	0.59 (0.52 to 0.65) *		
3 months	0.12 (0.05 to 0.18) ***p* < 0.001**	0.15 (0.09 to 0.22) ***p* < 0.001**	−0.03 (−0.11 to 0.03) *p* = 0.294	0.004
6 months	0.14 (0.08 to 0.20) ***p* < 0.001**	0.19 (0.13 to 0.26) ***p* < 0.001**	−0.05 (−0.12 to 0.02) *p* = 0.161	
12 months	0.19 (0.13 to 0.25) ***p* < 0.001**	0.19 (0.12 to 0.25) ***p* < 0.001**	0.00 (−0.07 to 0.07) *p* = 0.985	
BIPQ total score (0–80)	46.0 (43.2 to 48.8) *	43.9 (41.0 to 46.9) *		
3 months	−7.1 (−10.1 to −4.1) ***p* < 0.001**	−12.2 (−15.4 to −9.0) ***p* < 0.001**	5.1 (1.5 to 8.6) ***p* = 0.006**	0.007
6 months	−6.9 (−10.1 to −3.6) ***p* < 0.001**	−12.8 (−16.2 to −9.4) ***p* < 0.001**	6.0 (2.1 to 9.8) ***p* = 0.002**	
12 months	−9.3 (−12.7 to 5.9) ***p* < 0.001**	−13.2 (−16.7 to −9.6) ***p* < 0.001**	3.8 (−0.2 to 7.8) *p* = 0.060	
PEI (0–12)	Transition score, mean ± SE	Transition score, mean ± SE		
3 months	4.1 ± 0.3	5.4 ± 0.3	−1.4 (−2.3 to −0.4) ***p* = 0.005**	< 0.001
6 months	4.1 ± 0.4	5.6 ± 0.4	−1.6 (−2.7 to −0.4) ***p* = 0.007**	
12 months	4.3 ± 0.5	5.9 ± 0.5	−1.6 (−2.7 to −0.4) ***p* = 0.008**	

CPQI = clinical practice quality index, *n* = number of participants, SE = standard error, CI = confidence interval, ICC = intra-cluster correlation, ODI = Oswestry Disability Index, NRS = Numeric Rating Scale, EQ-5D = EuroQol 5 dimensions, BIPQ = Brief Illness Perception Questionnaire, PEI = Patient Enablement Instrument, PEI is transition rating scale with only 3, 6 and 12 months values. Bonferroni corrected significance thresholds of *p* ≤ 0.017 are printed in bold. * Mean (95% CI) at baseline.

**Table 7:** Comparisons of patient reported experience measures for patients receiving CPQI adherent/non adherent care.

	1. Non CPQI Adherent Care Group *n*/N (%)	2. CPQI Adherent Care Group *n*/N (%)	Between-Group Comparison (2/1), OR (95% CI), *p*-Value, ICC
**Patient satisfaction**			
Satisfied after 3 months	90/138 (65.2)	101/125 (80.8)	2.2 (1.3 to 4.0), ***p* = 0.006**, ICC = 0.006
Satisfied after 6 months	62/110 (56.4)	80/103 (77.7)	2.7 (1.5 to 6.4), ***p* = 0.001**, ICC = 0.002
Satisfied after 12 months	58/104 (55.8)	71/102 (69.6)	1.2 (1.0 to 3.2), *p* = 0.042, ICC < 0.001
**Patient global rating of change**			
Improved after 3 months	98/138 (71.0)	106/125 (84.8)	2.3 (1.2 to 4.2), ***p* = 0.009**, ICC < 0.001
Improved after 6 months	74/110 (67.3)	84/104 (80.8)	2.0 (1.1 to 3.9), *p* = 0.027, ICC < 0.001
Improved after 12 months	75/106 (70.8)	79/102 (77.5)	1.5 (0.8 to 2.8), *p* = 0.206, ICC = 0.035

*n* = number of participants with favorable outcome, N = total number of participants, CI = confidence interval, OR = odds ratio, ICC = intra-cluster correlation, CPQI = clinical practice quality index, LBP = low back pain. Bonferroni corrected significance thresholds of *p* ≤ 0.017 are printed in bold.

## Data Availability

The dataset used and/or analyzed during the current study are available from corresponding author on reasonable request.

## Supplementary Materials

The following are available online at https://www.mdpi.com/2077-0383/10/6/1230/s1, File S1.

## References

[1] Jordan K.P., Jöud A., Bergknut C., Croft P., Edwards J.J., Peat G., Petersson I.F., Turkiewicz A., Wilkie R., Englund M. (2014). International comparisons of the consultation prevalence of musculoskeletal conditions using population-based healthcare data from England and Sweden. Ann. Rheum. Dis..

[2] Ludvigsson M.L., Enthoven P. (2012). Evaluation of physiotherapists as primary assessors of patients with musculoskeletal disorders seeking primary health care. Physiotherapy.

[3] Goodwin R.W., Hendrick P.A. (2016). Physiotherapy as a first point of contact in general practice: A solution to a growing problem?. Prim. Health Care Res. Dev..

[4] Childs J.D., Fritz J.M., Wu S.S., Flynn T.W., Wainner R.S., Robertson E.K., Kim F.S., George S.Z. (2015). Implications of early and guideline adherent physical therapy for low back pain on utilization and costs. BMC Health Serv. Res..

[5] Oliveira C.B., Maher C.G., Pinto R.Z., Traeger A.C., Lin C.C., Chenot J.F., van Tulder M., Koes B.W. (2018). Clinical practice guidelines for the management of non-specific low back pain in primary care: An updated overview. Eur. Spine J..

[6] O’Connell N.E., Cook C.E., Wand B.M., Ward S.P. (2016). Clinical guidelines for low back pain: A critical review of consensus and inconsistencies across three major guidelines. Best Pract. Res. Clin. Rheumatol..

[7] Delitto A., George S.Z., Van Dillen L.R., Whitman J.M., Sowa G., Shekelle P., Denninger T.R., Godges J.J. (2012). Low back pain. J. Orthopaed. Sports Phys. Ther..

[8] Foster N.E., Anema J.R., Cherkin D., Chou R., Cohen S.P., Gross D.P., Ferreira P.H., Fritz J.M., Koes B.W., Peul W. (2018). Prevention and treatment of low back pain: Evidence, challenges, and promising directions. Lancet.

[9] Hartvigsen J., Hancock M.J., Kongsted A., Louw Q., Ferreira M.L., Genevay S., Hoy D., Karppinen J., Pransky G., Sieper J. (2018). What low back pain is and why we need to pay attention. Lancet.

[10] Forsetlund L., Bjorndal A., Rashidian A., Jamtvedt G., O’Brien M.A., Wolf F., Davis D., Odgaard-Jensen J., Oxman A.D. (2009). Continuing education meetings and workshops: Effects on professional practice and health care outcomes. Cochrane Database Syst. Rev..

[11] O’Brien M.A., Rogers S., Jamtvedt G., Oxman A.D., Odgaard-Jensen J., Kristoffersen D.T., Forsetlund L., Bainbridge D., Freemantle N., Davis D.A. (2007). Educational outreach visits: Effects on professional practice and health care outcomes. Cochrane Database Syst. Rev..

[12] Mesner S.A., Foster N.E., French S.D. (2016). Implementation interventions to improve the management of non-specific low back pain: A systematic review. BMC Musculoskelet. Disord..

[13] Berube M.E., Poitras S., Bastien M., Laliberte L.A., Lacharite A., Gross D.P. (2018). Strategies to translate knowledge related to common musculoskeletal conditions into physiotherapy practice: A systematic review. Physiotherapy.

[14] Scott S.D., Albrecht L., O’Leary K., Ball G.D., Hartling L., Hofmeyer A., Jones C.A., Klassen T.P., Kovacs Burns K., Newton A.S. (2012). Systematic review of knowledge translation strategies in the allied health professions. Implement. Sci..

[15] Bekkering G.E., van Tulder M.W., Hendriks E.J., Koopmanschap M.A., Knol D.L., Bouter L.M., Oostendorp R.A. (2005). Implementation of clinical guidelines on physical therapy for patients with low back pain: Randomized trial comparing patient outcomes after a standard and active implementation strategy. Phys. Ther..

[16] van der Wees P.J., Jamtvedt G., Rebbeck T., de Bie R.A., Dekker J., Hendriks E.J. (2008). Multifaceted strategies may increase implementation of physiotherapy clinical guidelines: A systematic review. Aust. J. Physiother..

[17] Stevenson K., Lewis M., Hay E. (2006). Does physiotherapy management of low back pain change as a result of an evidence-based educational programme?. J. Eval. Clin. Pract..

[18] Suman A., Dikkers M.F., Schaafsma F.G., van Tulder M.W., Anema J.R. (2016). Effectiveness of multifaceted implementation strategies for the implementation of back and neck pain guidelines in health care: A systematic review. Implement. Sci..

[19] Hanney W.J., Masaracchio M., Liu X., Kolber M.J. (2016). The Influence of Physical Therapy Guideline Adherence on Healthcare Utilization and Costs among Patients with Low Back Pain: A Systematic Review of the Literature. PLoS ONE.

[20] Abbott A., Schroder K., Enthoven P., Nilsen P., Oberg B. (2018). Effectiveness of implementing a best practice primary healthcare model for low back pain (BetterBack) compared with current routine care in the Swedish context: An internal pilot study informed protocol for an effectiveness-implementation hybrid type 2 trial. BMJ Open.

[21] Schröder K., Öberg B., Enthoven P., Kongsted A., Abbott A. (2020). Confidence, attitudes, beliefs and determinants of implementation behaviours among physiotherapists towards clinical management of low back pain before and after implementation of the BetterBack model of care. BMC Health Serv. Res..

[22] Hemming K., Haines T.P., Chilton P.J., Girling A.J., Lilford R.J. (2015). The stepped wedge cluster randomised trial: Rationale, design, analysis, and reporting. BMJ Clin. Res..

[23] Hooper R., Bourke L. (2014). The dog-leg: An alternative to a cross-over design for pragmatic clinical trials in relatively stable populations. Int. J. Epidemiol..

[24] Pinnock H., Barwick M., Carpenter C.R., Eldridge S., Grandes G., Griffiths C.J., Rycroft-Malone J., Meissner P., Murray E., Patel A. (2017). Standards for Reporting Implementation Studies (StaRI) Statement. BMJ Clin. Res..

[25] National Clinical Guideline Center (NICE) Low Back Pain and Sciatica in over 16s: Assessment and Management. http://www.nice.org.uk/guidance/gid-cgwave0681/documents.

[26] Sundhedsstyrelsen The National Clinical Guideline for Non-Surgical Treatment of Recently Lumbar Nervous System Impact (Lumbar Radiculopathy) Provides Recommendations on Non-Surgical Treatment Options. http://sundhedsstyrelsen.dk/da/udgivelser/2016/lumbal-nerverodspaavirkning-ikke-kirurgisk-behandling.

[27] Sundhedsstyrelsen National Clinical Guidelines for Non-Surgical Treatment of Newly Occurring Lower Back Pain. http://sundhedsstyrelsen.dk/da/udgivelser/2016/nkr-laenderygsmerer.

[28] Michie S., van Stralen M.M., West R. (2011). The behaviour change wheel: A new method for characterising and designing behaviour change interventions. Implement. Sci..

[29] Michie S., Johnston M., Francis J., Hardeman W., Eccles M. (2008). From theory to intervention: Mapping theoretically derived behavioural determinants to behaviour change techniques. Appl. Psychol..

[30] Michie S., Johnston M. (2004). Changing clinical behaviour by making guidelines specific. BMJ Clin. Res..

[31] Briggs A.M., Jordan J.E., Jennings M., Speerin R., Chua J., Bragge P., Slater H. A Framework to Evaluate Musculoskeletal Models of Care. Cornwall: Global Alliance for Musculoskeletal Health of the Bone and Joint Decade. https://www.aci.health.nsw.gov.au/__data/assets/pdf_file/0020/338141/Framework-to-Evaluate-Musculoskeletal-MoC.pdf.

[32] Leventhal H., Phillips L.A., Burns E. (2016). The Common-Sense Model of Self-Regulation (CSM): A dynamic framework for understanding illness self-management. J. Behav. Med..

[33] Hoffmann T.C., Glasziou P.P., Boutron I., Milne R., Perera R., Moher D., Altman D.G., Barbour V., Macdonald H., Johnston M. (2014). Better reporting of interventions: Template for intervention description and replication (TIDieR) checklist and guide. BMJ Clin. Res..

[34] Jensen M.P., Turner J.A., Romano J.M., Fisher L.D. (1999). Comparative reliability and validity of chronic pain intensity measures. Pain.

[35] Fairbank J.C., Pynsent P.B. (2000). The Oswestry Disability Index. Spine.

[36] Goldsmith E.S., Taylor B.C., Greer N., Murdoch M., MacDonald R., McKenzie L., Rosebush C.E., Wilt T.J. (2018). Focused Evidence Review: Psychometric Properties of Patient-Reported Outcome Measures for Chronic Musculoskeletal Pain. J. Gen. Intern. Med..

[37] Chapman J.R., Norvell D.C., Hermsmeyer J.T., Bransford R.J., DeVine J., McGirt M.J., Lee M.J. (2011). Evaluating common outcomes for measuring treatment success for chronic low back pain. Spine.

[38] The EuroQol Group (1990). EuroQol—A new facility for the measurement of health-related quality of life. Health Policy.

[39] Broadbent E., Petrie K.J., Main J., Weinman J. (2006). The brief illness perception questionnaire. J. Psychosom. Res..

[40] Roost M., Zielinski A., Petersson C., Strandberg E.L. (2015). Reliability and applicability of the Patient Enablement Instrument (PEI) in a Swedish general practice setting. BMC Fam. Pract..

[41] Kamper S.J., Maher C.G., Mackay G. (2009). Global rating of change scales: A review of strengths and weaknesses and considerations for design. J. Man. Manip. Ther..

[42] Butler R.J., Johnson W.G. (2008). Satisfaction with low back pain care. Spine J. Off. J. N. Am. Spine Soc..

[43] Cieza A., Stucki G., Weigl M., Disler P., Jackel W., van der Linden S., Kostanjsek N., de Bie R. (2004). ICF Core Sets for low back pain. J. Rehab. Med..

[44] Revicki D., Hays R.D., Cella D., Sloan J. (2008). Recommended methods for determining responsiveness and minimally important differences for patient-reported outcomes. J. Clin. Epidemiol..

[45] van der Roer N., Ostelo R.W., Bekkering G.E., van Tulder M.W., de Vet H.C. (2006). Minimal clinically important change for pain intensity, functional status, and general health status in patients with nonspecific low back pain. Spine.

[46] Soer R., Reneman M.F., Vroomen P.C., Stegeman P., Coppes M.H. (2012). Responsiveness and minimal clinically important change of the Pain Disability Index in patients with chronic back pain. Spine.

[47] Hosmer D.W., Lemeshow S. (2000). Applied Logistic Regression.

[48] Fox-Wasylyshyn S.M., El-Masri M.M. (2005). Handling missing data in self-report measures. Res. Nurs. Health.

[49] Enthoven P., Eddeborn F., Abbott A., Schröder K., Fors M., Öberg B. (2021). Patients’ experiences of the BetterBack model of care for low back pain in primary care—A qualitative interview study. Int. J. Qual. Stud. Health Well Being.

[50] Bamm E.L., Rosenbaum P., Wilkins S. (2013). Is Health Related Quality of Life of people living with chronic conditions related to patient satisfaction with care?. Disab. Rehab..

[51] Al Zoubi F.M., Menon A., Mayo N.E., Bussières A.E. (2018). The effectiveness of interventions designed to increase the uptake of clinical practice guidelines and best practices among musculoskeletal professionals: A systematic review. BMC Health Serv. Res..

[52] Beneciuk J.M., George S.Z. (2015). Pragmatic Implementation of a Stratified Primary Care Model for Low Back Pain Management in Outpatient Physical Therapy Settings: Two-Phase, Sequential Preliminary Study. Phys. Ther..

[53] Cherkin D., Balderson B., Wellman R., Hsu C., Sherman K.J., Evers S.C., Hawkes R., Cook A., Levine M.D., Piekara D. (2018). Effect of Low Back Pain Risk-Stratification Strategy on Patient Outcomes and Care Processes: The MATCH Randomized Trial in Primary Care. J. Gen. Intern. Med..

[54] Masterson-Algar P., Burton C.R., Rycroft-Malone J., Sackley C.M., Walker M.F. (2014). Towards a programme theory for fidelity in the evaluation of complex interventions. J. Eval. Clin. Pract..

[55] Rycroft-Malone J. (2015). It’s more complicated than that Comment on “Translating evidence into healthcare policy and practice: Single versus multi-faceted implementation strategies—Is there a simple answer to a complex question?”. Int. J. Health Policy Manag..

[56] Hoeijenbos M., Bekkering T., Lamers L., Hendriks E., van Tulder M., Koopmanschap M. (2005). Cost-effectiveness of an active implementation strategy for the Dutch physiotherapy guideline for low back pain. Health Policy.

[57] Shenoy S. (2013). Cluster Randomized Controlled Trial to Evaluate the Effectiveness of a Multifaceted Active Strategy to Implement Low Back Pain Practice Guidelines: Effect on Competence, Process of Care and Patient Outcome in Physical Therapy. Ph.D. Thesis.

[58] Foster N.E. (2011). Barriers and progress in the treatment of low back pain. BMC Med..

[59] Evans D.W., Breen A.C., Pincus T., Sim J., Underwood M., Vogel S., Foster N.E. (2010). The effectiveness of a posted information package on the beliefs and behavior of musculoskeletal practitioners: The UK Chiropractors, Osteopaths, and Musculoskeletal Physiotherapists Low Back Pain ManagemENT (COMPLeMENT) randomized trial. Spine.

[60] Bekkering G.E., Hendriks H.J., van Tulder M.W., Knol D.L., Hoeijenbos M., Oostendorp R.A., Bouter L.M. (2005). Effect on the process of care of an active strategy to implement clinical guidelines on physiotherapy for low back pain: A cluster randomised controlled trial. Qual. Saf. Health Care.

[61] Fritz J.M., Cleland J.A., Speckman M., Brennan G.P., Hunter S.J. (2008). Physical therapy for acute low back pain: Associations with subsequent healthcare costs. Spine.

[62] Ostelo R.W., Deyo R.A., Stratford P., Waddell G., Croft P., Von Korff M., Bouter L.M., de Vet H.C. (2008). Interpreting change scores for pain and functional status in low back pain: Towards international consensus regarding minimal important change. Spine.

[63] Hurwitz E.L., Morgenstern H., Yu F. (2005). Satisfaction as a predictor of clinical outcomes among chiropractic and medical patients enrolled in the UCLA low back pain study. Spine.

[64] Meucci R.D., Fassa A.G., Faria N.M. (2015). Prevalence of chronic low back pain: Systematic review. Rev. Saude Publica.

[65] Öberg B., Enthoven P., Kjellman G., Skargren E. (2003). Back pain in primary care: A prospective cohort study of clinical outcome and healthcare consumption. Adv. Physiother..

[66] Boissoneault J., Mundt J., Robinson M., George S.Z. (2017). Predicting Low Back Pain Outcomes: Suggestions for Future Directions. J. Orthopaed. Sports Phys. Ther..

[67] Fritz J.M., Magel J.S., McFadden M., Asche C., Thackeray A., Meier W., Brennan G. (2015). Early Physical Therapy vs Usual Care in Patients with Recent-Onset Low Back Pain: A Randomized Clinical Trial. JAMA.

[68] Bier J.D., Sandee-Geurts J.J.W., Ostelo R., Koes B.W., Verhagen A.P. (2018). Can Primary Care for Back and/or Neck Pain in the Netherlands Benefit from Stratification for Risk Groups According to the STarT Back Tool Classification?. Arch. Phys. Med. Rehab..

[69] Enthoven P., Skargren E., Oberg B. (2004). Clinical course in patients seeking primary care for back or neck pain: A prospective 5-year follow-up of outcome and health care consumption with subgroup analysis. Spine.

[70] Folkhälsomyndigheten Open Comparisons Public Health. https://www.folkhalsomyndigheten.se/publicerat-material/publikationsarkiv/oe/oppna-jamforelser-folkhalsa-2019/.

[71] Moore G.F., Audrey S., Barker M., Bond L., Bonell C., Hardeman W., Moore L., O’Cathain A., Tinati T., Wight D. (2015). Process evaluation of complex interventions: Medical Research Council guidance. BMJ Clin. Res..

