# Supplementary material for: Effectiveness and Quality of Implementing a Best Practice Model of Care for Low Back Pain (BetterBack) Compared with Routine Care in Physiotherapy: A Hybrid Type 2 Trial
Source: J Clin Med. 2021 Mar 16;10(6):1230. doi: 10.3390/jcm10061230 (PMC8002355; doi:10.3390/jcm10061230)
Supplement: Supplementary file 1 [file jcm-10-01230-s001.pdf]

BetterBack☺ Model of care for LBP

<u>Östergötland health care region physiotherapeutic clinical practice guideline recommendations for primary care management of benign LBP with or without radiculopathy</u>

Each evidence based guideline recommendation is supported by a clinical priority ranking. This is based on an overall assessment of the severity of the condition, reported effect of the intervention, strength of evidence assessment (GRADE), cost-effectiveness and the benefit of the intervention based on professional experience and patient benefit. A scale from 1 to 10 is used where the number 1 indicates recommended practices with the highest priority while the number 9 indicates recommended practices of low priority. The number 10 indicates recommendations that provide very little or no benefit or utility and are therefore not recommended.

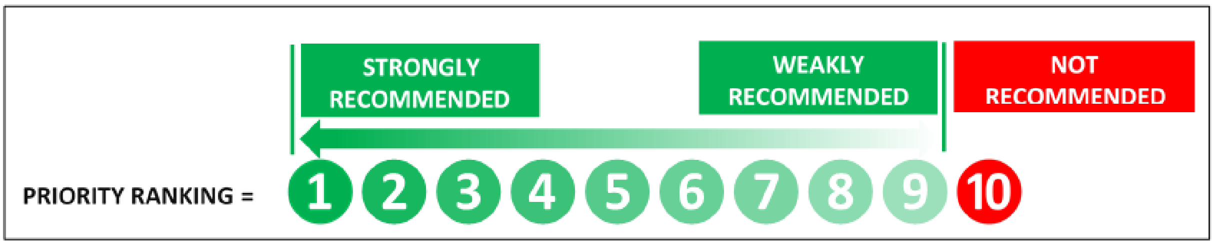

**Recommendation 1**	**PRIORITY RANKING =** 1 **(2)** 3 4 5 6 7 8 9 10

Routine care should consist of standardised processes for subjective and objective assessment and diagnostics. A thorough screening of red flags is essential to rule out serious pathology. Treatment should be individualised for each patient. Basic treatment principles should be based on reassurance of a good prognosis, maintenance of appropriate physical activity and self-care enablement.
Justification: The work group's reasoning is based on clinical experience of the importance of careful screening to rule out serious pathology. Furthermore, standardised assessment and diagnostics provide quality assurance but treatment needs to be individualised for each patient case. The work group also reasoned based on clinical experience that appropriate physical activity is likely to contribute to maintaining the patient's functional level, psychosocial and general health as well as have positive effects on self-care enablement. In some cases, may physical activity temporarily aggravate pain and symptoms, but there are no known persisting side effects. The work groups reasoning is also based on evidence showing a statistically significant advantage for maintaining appropriate physical activity compared to bed rest for improving pain and function. Despite this, evidence that proves the benefit of appropriate physical activity is so great to be clinically relevant is missing. In addition, the best available evidence has however a currently limited scientific basis (⊗⊗OO). *<u>The working group proposes the following resources in the BetterBack☺ model of care to support the implementation of Recommendation 1 (See sections 1-5)</u>*

**Recommendation 2**	**PRIORITY RANKING =** 1 2 3 4 5 6 7 8 9 **(10)**

Do not perform routine medical imaging investigations (eg X-ray, CT, MRI)
Justification: The work group´s reasoning is based on evidence that shows no differences in outcomes of pain, function and quality of life between patients who received or did not receive

©Linköping University 01042017

routine medical imaging investigations in the primary care context. The best available evidence has however a currently inadequate scientific basis (⊗○○○). It was also discussed that imaging cannot confirm or reject a preliminary diagnosis as the relationship between patient symptoms and degenerative imaging finding is usually weak. Moreover, degenerative secondary findings are common in asymptomatic individuals. *The work group however suggests that early use of medical imaging is motivated in the presence of symptoms or signs suggesting possible serious underlying pathology (red flags). Medical imaging may also be relevant when pain persists despite primary care treatment.*

Recommendation 3	PRIORITY RANKING = 1 2 3 4 5 **6** 7 8 9 10

Consider using a patient-reported tool (eg STarT Back risk assessment tool) as usual care during the early-stages of patient management to screen the risk of continued LBP

Justification: The work group's reasoning is based on studies showing that STarT Back Tool is the only valid tool to investigate the risk of continued back pain in the primary care context. It shows the highest accuracy for detecting patients with low risk profile (total score ≤3) and medium-high risk profile (total score ≥4) for continued back pain. Studies also show that STarT Back Tool has the best ability to predict functional and pain-related outcomes. The best available evidence has however a currently inadequate scientific basis (⊗○○○). No economical evaluations were identified but the working group discussed the importance of a simple and fast tool. STarT Back Tool can be filled in and analyzed in a few minutes to advantage over other tools that can be an administrative burden for patients and healthcare professionals. *The working group argues that the predictive value of the tool should support, but not replace, regular examination procedures and clinical decision making. See section 3 for STarT Back Tool.*

Recommendation 4	PRIORITY RANKING = 1 2 3 4 5 6 7 **8** 9 10

Consider using a patient-reported tool (such as the STarT Back risk assessment tool) and classification of examination findings during the early-stages of patient management to aid the stratification of care to prevent continued LBP

Justification: The work group reasoned that for the choice and scope of targeted treatment measures, consideration should be given to the assessment of risk profile for long-term LBP and classification of examination findings. This has been shown to have a better effect on pain, function and quality of life, as well as less economic costs compared to no treatment stratification. The best available evidence has however a currently inadequate scientific basis (⊗○○○). For a patient with low risk profile (total score ≤3 on STarT Back Tool) usual care is relevant and requires only few visits, but the working group recommends that adequate treatment measures directed at examination findings is of the highest importance. For patients with medium-high risk profile (total score ≥ 4 on STarT Back Tool), usual care will require additional visits. Information provided in questions 5-9 on STarT Back Tool that investigate anxiety with psychological risk factors can guide the need, focus and extent of behavioral medicine measures. *The working group argues that stratified care classified after assessing a risk profile for long-term back pain should support but not replace conventional examination procedures and clinical decision-making for treatment measures. The working group proposes the following resources to support the implementation of targeted treatments based on stratification (See sections 1-5).*

Recommendation 5	PRIORITY RANKING = 1 2 3 **4** 5 6 7 8 9 10

Consider giving individualised patient education as a part of usual care (e.g. an explanatory model based on pain neuroscience and psychological mechanisms)

©Linköping University 01042017

Justification: Based on the best available evidence, the work group reasoned that individualised patient education as part of usual care can result in reduced work sickness absenteeism. The priority of the recommendation has been strengthened by consensus within the work group based on proven experience that individual adapted patient education is an important part of patient-centered care. The best available evidence has however a currently inadequate scientific basis (⊗○○○). The intervention requires that the patient is receptive for education. The extent of patient education can depend upon whether the patient has a distorted image of the underlying mechanism of LBP and a high degree of negative outcome expectations, anxiety, and fear-avoidance or if they are inactive or passive in managing the LBP. Patient education should include a reassuring dialogue and other cognitive and behavioural therapeutic techniques of relevance to support change in the individual's maladaptive thoughts, feelings and behaviors. Pedagogical explanation models should be used to provide the patient with knowledge about symptoms and disorders, as well as to strengthen and support self-care ability to master everyday activities. *The work group proposes the following resources to support of the implementation of patient education (See sections 6-7)*

**Recommendation 6**	**PRIORITY RANKING =** 1 2 3 **4** 5 6 7 8 9 10

Consider a supervised exercise program as part of usual care
Justification: Supervised training is defined as general or back-specific exercises or physical activities conducted under the guidance of a healthcare professionals. The work group's reasoning is based on scientific evidence and proven experience that supervised training as part of usual care can result in clinically relevant improvement in pain, function, quality of life and produces lower health care costs compared with no supervised training. There is however no evidence that a specific type of exercise would be superior to another. The best available evidence has however a currently limited scientific basis (⊗⊗○○).
The work group proposes the following resources to support the implementation of a supervised training program (see section 8).

**Recommendation 7**	**PRIORITY RANKING =** 1 2 3 **4** 5 6 7 8 9 10

Consider mobilisation techniques for neuromusculoskeletal structures as part of usual care (including active or passive motion in an angular and / or translational plane)
Justification: The working group reasoning is based on evidence that for patients with segmental movement impairments, mobilization techniques can provide a statistically significant reduction in short-term pain. It is however uncertain whether the effect is sufficiently large so that patients experience a clear improvement overtime. At group level, there is no evidence that a particular technique is be superior to another. It cannot be ruled out that for subgroups of LBP patients, more positive effects on pain and function may be produced by specific mobilisation techniques. It is expected that these subgroups can be identified by careful diagnostics and short trial treatments. Mobilizing techniques as part of multimodal treatment provide better results. Serious side effects are rare. However, the best available evidence is based on a currently limited scientific basis (⊗⊗○○).

**Recommendation 8**	**PRIORITY RANKING =** 1 2 3 4 5 6 7 8 **9** 10

Consider acupuncture treatment in addition to usual care
Justification: The working group reasoned based on evidence that cannot exclude acupuncture has a short-term pain relief effect in addition to a placebo effect. Acupuncture has however no effect on function. Side effects in the form of brief superficial bleeding or inflammation may occur.

©Linköping University 01042017

Pneumothorax and systemic infections are not common, but the prevalence is unknown. The best available evidence has however a currently inadequate scientific basis (⊗○○○).

Recommendation 9	PRIORITY RANKING = 1 2 3 4 5 6 7 8 9 10

Do not offer corset, shoes, traction, ultrasound or electrotherapy
Justification: The work group's reasoning is based on evidence that passive treatments such as corset, shoots / soles, traction, ultrasound or electrotherapy do not reduce pain or improve function and quality of life in patients more than no treatment or when offered as part of multimodal treatment. However, the best available evidence is based on a currently limited scientific basis (⊗⊗○○). ***It cannot be ruled out that subgroups of patients may experience positive effects of these interventions when a hypothesised effect mechanism is aimed at specific functional impairment or activity limitation.***

Recommendation 10	PRIORITY RANKING = 1 2 3 4 5 6 7 8 9 10

Consider prescription-free NSAID medication if necessary in addition to usual treatment (lowest dose and shortest possible treatment time).
NSAIDs: There is evidence of the effect of NSAID in patients with long-term LBP but the effect has not been highlighted on short-term pain or functional outcomes. There are no adverse reactions reported in systematic review studies on LBP, but potential transient side effects of NSAIDs such as reduced blood clotting, reduced stomach mucous function and reduced kidney function are known from studies on other conditions. The work group reasoned that lowest dose and shortest possible treatment time decreases the risk of side-effects. The work group anticipates that there are differences in patient preferences regarding NSAIDs, where some patients will agree to NSAID treatment, while others will decline. The best available evidence for NSAID effects on LBP outcomes is based on an inadequate scientific evidence (⊗○○○). The work group reasoned based on clinical experience that it cannot be excluded that the NSAID may have a pain relief effect in the short term.

Recommendation 11	PRIORITY RANKING = 1 2 3 4 5 6 7 8 9 10

Do not offer paracetamol or opioids
Paracetomol: Has no effect on the degree of LBP and functional ability. There are no reported adverse reactions in studies, but side effects of paracetamol in the form of hepatic effects are known from studies on other conditions. The best available evidence is based on a moderately strong scientific basis (⊗⊗⊗○).

Opioids: A weak analgesic effect of oxycodone in combination with paracetamol has been demonstrated in a study but the intervention has no effect on functional capacity for up to 12 weeks. Other positive effects or adverse effects were not shown. A wide range of opioid side effects are known from other studies. Therefore, the working group reasoned that treatment results in more risks than benefits to the patient. The best available evidence is based on a currently limited scientific basis (⊗⊗○○).

©Linköping University 01042017

BetterBack☺ model of care implementation support tools

1. Subjective assessment proformer for therapist use

LOW BACK SUBJECTIVE ASSESSMENT PROFORMER

Name:…………………………………………………… Date of birth:……………………………………………..
Date:…………………………………………..

**History of the present condition** (debut, duration, activity limitation)	**Symptom localisation**<br>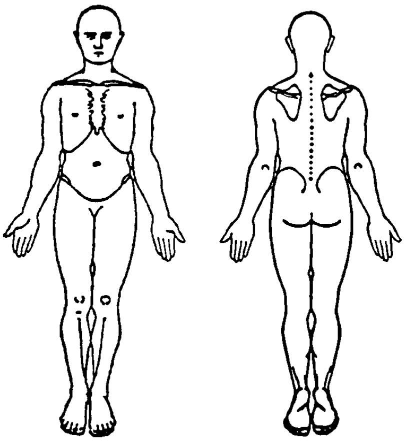

**Symptom Description**	Localisation back	Localisation right leg	Localisation left leg
Pain nature (Dull, stabbing, radiating etc)			
Pain frequency (Constant/ Intermittent)			
Pain Intensity (NRS 0-10)			
Daily variation (am/pm, night time pain/disturbed sleep)			
Irritability (non-irritable/highly irritable)			
Aggravating factors (loading etc)			
Easing faktors (rest etc)			
Course (Improving/same/worse)			
Other symptoms (Instability, weakness, paresthesia, stiffness)			

**Past medical history**<br>Previous level of function/activity:<br><br>Previous treatment:	Red flags: (malignancy, unexplained weight loss, trauma, osteoporosis, infection, inflammatory disease, spinal cord compression symtoms, drug use)<br><br>Other illnesses/ General health:
**Work, Social, Family history**	**Patient förväntningar**
**Medication**	**Medical imaging/Laboratory tests**

©Linköping University 01042017

2. Physical assessment proformer

LOW BACK PHYSICAL ASSESSMENT PROFORMER

1. INSPECTION – Postural screen

Sitting: good/fair/poor	Postural correction: Better/Worse/No effect	
Standing: good/fair/poor	Postural correction: Better/Worse/No effect	
Lordosis: Hyper/hypo/normal	Kyphosis: Hyper/hypo/normal	Lateralt shift: Right/Left/none
Spinal symmetry:	Shoulder symmetry:	Pelvic symmetry:
Leg & fot symmetry:	Muscular hypo/hypertrophy:	Scars:

2. SCREENING OF FUNCTIONAL MOVEMENT:

Shoes on/off, sit-stand, 2 leg/ 1 leg squat, lunge right/left

Gait: Trendelenburg right/left
- Limp right/left
- Weight transfer right/left
- Toe walking right/left
- Heel walking right/left

Work or sport specific:________________________________

3. SCREENING TEST IN STANDING/SITTING

	Right	Left
Slump test + sensitisation head/foot		
Foramen compression/unloading		
Hip loading/unloading in standing		

4. TEST IN STANDING/SITTING
LUMBAR ACTIVE ANGULAR MOVEMENT

	Range			Quality		Symptoms		
	Large	Med	Small	High	Low	During range	End range	Rep Mov
Flex								
Ext								
Lateral flex	R L	R L	R L	R L	R L	R L	R L	R L
Side Glide	R L	R L	R L	R L	R L	R L	R L	R L
Rot	R L	R L	R L	R L	R L	R L	R L	R L
Coupled flex	R L	R L	R L	R L	R L	R L	R L	R L
Coupled ext	R L	R L	R L	R L	R L	R L	R L	R L

5. TEST IN SIDE LYING
LUMBAR PASSIVE ANGULAR MOVEMENT

	Range			Symptoms			
	Large	Med	Small	During range	End range	Rep Mov	Over press
Flex							
Ext							
Lat flex	R L	R L	R L	R L	R L	R L	R L
Rot	R L	R L	R L	R L	R L	R L	R L
Coupled flex	R L	R L	R L	R L	R L	R L	R L
Coupled ext	R L	R L	R L	R L	R L	R L	R L

6. PRONE
ACCESSORY MOVEMENT/NERVE & MUSCLE FUNCTION

Spinal extension in prone	Better/Worse/No effect			
Segmental provocation	Movement			Pain
	Hyper	Hypo	Normal	
- Central P/A, Springing test<br>- Unilateral P/A<br>- Rotation provocation<br>- Prone instability test				
Femoral nerve tension test				
Isometric/dynamic back muscle tests				

7. SUPINE
DIFFERENTIAL DIAGNOSTICS HIP/SI-JOINT/BACK

Spinal flexion in supine	Better/Worse/No effect	
Isometric/dynamic abdominal muscle tests		
	Right	Left
Hip: Angular movement, Patricks test, quadrant		
SI-joint provocation test, ASLR		
Passive SLR + head/foot sensitisation, crossed SLR		
Myotomes- L1-2(I), L2-3(Q), L4-5(TA), L5(EH), L5-S1(P), S1(TS)		
Dermatomes		
Reflexs: Patella L3-4, Achilles S1		
Babinski, Klonus		

8. PALPATION

©Linköping University 01042017

3. STarT Back Tool

Patient name: ______________________________ Date: ____________

Thinking about the **last 2 weeks** tick your response to the following questions:

		Disagree 0	Agree 1
1	My back pain has **spread down my leg(s)** at some time in the last 2 weeks	☐	☐
2	I have had pain in the **shoulder** or **neck** at some time in the last 2 weeks	☐	☐
3	I have only **walked short distances** because of my back pain	☐	☐
4	In the last 2 weeks, I have **dressed more slowly** than usual because of back pain	☐	☐
5	It's not really safe for a person with a condition like mine to be physically active	☐	☐
6	**Worrying thoughts** have been going through my mind a lot of the time	☐	☐
7	I feel that **my back pain is terrible** and **it's never going to get any better**	☐	☐
8	In general I have **not enjoyed** all the things I used to enjoy	☐	☐

9. Overall, how **bothersome** has your back pain been in the **last 2 weeks**?

Not at all	Slightly	Moderately	Very much	Extremely
☐	☐	☐	☐	☐
0	0	0	1	1

Total score (all 9): ________________ **Sub Score (Q5-9):**_____________

© Keele University 01/08/07
Funded by Arthritis Research UK

©Linköping University 01042017

4. Clinical Reasoning and Process Evaluation tool (CRPE-tool) for therapists

**PATIENT NAME:________________________**<br>**DATE OF BIRTH:________________________**	**First assessment date:___/___/___**<br>**Final assessment date:___/___/___**<br>**Totalt number of physiotherapy visits:_________**

ASSESSMENT

- **First assessment - cross X relevant assessment findings**
- **Final assessment - circle ○ relevant assessment findings**

**1. Assess grade of FUNCTIONAL IMPAIRMENT**	**None**	**Lite**	**Moderate**	**Severe**	**Complete**	KVÅ code
Energy and drive (motivation)	0	1	2	3	4	PA006
Sleep functions	0	1	2	3	4	PA007
Emotional functions (anxiety, low mood)	0	1	2	3	4	PA011
Thought functions (physical symptoms caused by cognitive/rational factors)	0	1	2	3	4	PA013
Sensory function (sensitivity for pain ”sensitisation”)	0	1	2	3	4	PB008
Pain (choose relevant category)						
Back pain	0	1	2	3	4	PB009
Lower extremity pain	0	1	2	3	4	PB009
Pain in a dermatome	0	1	2	3	4	PB009
Pain in another body part (Buttock, hip, groin, thigh)	0	1	2	3	4	PB009
Generalised pain localisation (3 of 4 body quadrats)	0	1	2	3	4	PB009
Exercise tolerance (endurance related activities)	0	1	2	3	4	PD009
Joint mobility	0	1	2	3	4	PG001
Joint stability	0	1	2	3	4	PG002
Muscle power	0	1	2	3	4	PG003
Muscle tone	0	1	2	3	4	PG003
Muscle endurance	0	1	2	3	4	PG003
Motor reflex funktions (decreased or increased)	0	1	2	3	4	PG004
Control of movement (Quality, coordination, balance)	0	1	2	3	4	PG006
Gait pattern	0	1	2	3	4	PG007
Sensation of muscle stiffness, tightness, spasm, contraction, heaviness	0	1	2	3	4	PG003
Mobility of spinal meningies, periferal nerves and surrounding tissue	0	1	2	3	4	PG000
**2. Assess grade of ACTIVITY LIMITATION**	**None**	**Lite**	**Moderate**	**Severe**	**Complete**	KVÅ code
Perception of non-harmful sensory stimuli (kinesiophobia)	0	1	2	3	4	PJ001
Carrying out daily routine (ADL)	0	1	2	3	4	PK003
Handling stress and other psychological demands	0	1	2	3	4	PK004
Changing and maintaining body position (Shifting body weight away from the spine (increased lever arm)	0	1	2	3	4	PM001
Changing and maintaining body position (bending)	0	1	2	3	4	PM001
Maintaining a lying position	0	1	2	3	4	PM001
Maintaining a sitting position	0	1	2	3	4	PM001
Maintaining a standing position	0	1	2	3	4	PM001
Maintaining an upright neutral posture	0	1	2	3	4	PM001
Lyfting and carrying objects	0	1	2	3	4	PM004
Walkning	0	1	2	3	4	PM007

©Linköping University 01042017

Moving around in different ways (crawling/climbing, running/joging, jumping)	0	1	2	3	4	PM008
Household tasks	0	1	2	3	4	PP003
Work ability and employment	0	1	2	3	4	PR002
Recreation and leisure activities	0	1	2	3	4	PS002

DIAGNOSTIC SUBGROUPING AND ICD-10 CODING

3. Matching assessment findings to diagnostic codes
Choose a primary assessment finding category:

- **First assessment: Cross X one or more related ICD-10 diagnostic codes in the same row**
- **Final assessment: Circle ○ a new diagnostic codes <u>if relevant</u>.**

**Primary assessment category** →	**ICD-10 diagnos**
LBP with muscular functional impairment	☐ M54.5 Lumbago
LBP with segmental mobility impairment	☐ M54.5 Lumbago<br>☐ M99.0 Segmental dysfunction
LBP with movement coordination impairment/ segmental instability	☐ M54.5 Lumbago<br>☐ M99.1K Segmental instability in the lumbar spine
LBP with referred lower extremity pain (nociceptive pain proximal of the knee)	☐ M54.5 Lumbago<br>☐ M51.2 Other specificed dislocation of intervertebral disc<br>☐ M47.9K Spondylosis in the lumbar spine
LBP with radiating pain (neuropathic pain)	☐ M54.5 Lumbago<br>☐ M54.1 Radiculopathy (femoralis)<br>☐ M54.4 Lumbago with ischias
LBP with related cognitive or affective tendensies	☐ M54.5 Lumbago<br>☐ G96.8 Other specified disorders of the CNS (pain sensitivity)
LBP with related generaliserad pain (pain in 3 of 4 body quadrants)	☐ M54.5 Lumbago<br>☐ G96.8 Other specified disorders of the CNS (pain sensitivity)<br>☐ F45.4 Chronic somatoform pain syndrome
LBP with postural related symptoms	☐ M54.5 Lumbago<br>☐ M40.3 Flatback syndrome<br>☐ M40.4 Hyperlodosis
SI-joint symptoms or Coccygodynia	☐ M53.3 Sacrococcygeal disorders
LBP radiating pain + Medical imaging disc pathology and nerve compression finding	☐ M51.1K Disc degeneration/disc herniation in the lumbar spine with radiculopathy
LBP with radiating pain/neurogenic claudication + Medical imaging verifieried degeneration and nerve compression findings	☐ M48.0K Central spinal stenos in the lumbar spine (bilateral symptoms)<br>☐ M99.6 Stenosis of intervertebral foramin (unilateralt symptoms)

©Linköping University 01042017

Ländryggsbesvär med nedsatt rörelse kontroll i ryggen och/eller segmentell instabilitet + Medicinsk bild verifierad Spondylolys/Spondylolisthes	☐ M43.0 Spondylolys<br>☐ M43.1 Spondylolistes

TREATMENT

4. Record at final assessment:

Has the BetterBack☺ model of care Part 1 been applied?		☐ Yes ☐ No
Has the BetterBack☺ model of care Part 2 been applied?		☐ Yes ☐ No
Cross X all modes och types of treatments used		
Physical exercise	**MODE**	KVÅ code
	☐ Non-supervised individual training	
	☐ Supervised individual training	QV011
	☐ Supervised group training	QV012
	**TYPE**	
	☐ Muscle strengthening training	QG003
	☐ Range of movement training	QG001
	☐ Muscle endurance training	QG003
	☐ Cardiovascular training	QD016
	☐ Balance training	QB001
	☐ Postural control training	QG004
	☐ Coordination training	QG005
	☐ Pelvic floor training	QF001
	☐ Postural training	QM005
	☐ Relaxation training	QG007
	☐ Physical activity prescription (FaR®)	DV002
	☐ Other	
Behavioural medicine interventions	**MODE**	
	☐ Individual based intervention	QV011
	☐ Group based intervention	QV012
	**TYPE**	
	☐ Information / education on pain	QV007
	☐ Cognitive-behavioural therapy	DU011
	☐ Mindfulness	DU032
	☐ Motivational interviewing	DU118
	☐ Relapse prevention	DU119
	☐ Supportive conversation	DU007
	☐ Other	
Manual therapy	**TYPE**	
	☐ Joint mobilisation	DN006
	☐ Joint manipulation	DN008
	☐ Massage	QB007
	☐ Stretching	DN009
	☐ Nerve mobiliseration	QG001
	☐ Trigger point pressure	DN007
	☐ Traction	QG001
	☐ Other.............................	
Occupational medicine interventions	**TYPE**	
	☐ Workplace training	DV084
	☐ Training of work ability	QR003
	☐ Work and employment counciling	QR002
	☐ Information /education on ergonomics	QV010
	☐ Other	
Physical modalities	**TYPE**	
	☐ TENS	DA021
	☐ Cryotherapy	QB011
	☐ Heat	QB011
	☐ Ultrasound	QB011
	☐ Shockwave therapy	QB011

©Linköping University 01042017

	☐ Laser therapy	QB011
	☐ Short wave diathermy	DV042
	☐ Interferential therapy	DA021
	☐ Orthosis	DN003
	☐ Taping	DN003
	☐ Bio-feedback	DV010
	☐ Acupunkture	DA001
	☐ Other…………………………	
**5. Rate overall treatment effect**	☐ Much better<br>☐ Quite much better<br>☐ Unchanged<br>☐ Quite much worse<br>☐ Much worse	

5. Clinical reasoning and process pathway for therapists

A thorough history and adequate physical examination are of great importance in order to target treatment interventions. In addition, it is very important to exclude the few red flag cases that require acute medical or specialist referral for the investigation and treatment of tumors, infections, inflammatory diseases, more severe back pathology and neurological conditions, as well as the strong influence of psychosocial factors which can also cause back pain. StarT Back Tool can be used to support decision making regarding the extent of health care needed and the need for psychosocial focus based on an assessment of risk factors for continued back pain. The physical assessment should include an analysis of functional movements, posture, active movements, passive movements, combined movements and / or static positions, joint accessory movement / provocation tests and neuromuscular function. This is to investigate how the symptoms are related to motion dysfunction.

Based on assessment findings, relevant treatment measures with effect mechanisms directed at functional impairments and activity limitations should be tested. These may include range of movement exercises (active/passive or accessory joint mobilisation or neuromuscular structure mobilisation), motor control exercises, muscle stretching, balance exercises, coordination, muscle strength, muscle endurance, general physical fitness or cardiovascular exercise. For example:

1. In the identification of movement directions and positions that reduce or centralize the patient's localised pain, distal pain or radiculopathy, these may be considered as a treatment techniques. This allows the patient to learn strategies to control pain and thus take better responsibility for his or her own situation.

2. In the identification of movement restriction due to joint, muscle or nerve related impairment, mobilisation strategies for the relevant structure may be considered to reduce the movement restriction.

3. In the identification of segmental instability or trunk motor control impairment in the, exercises with a focus on movement control can be tested aiming to improve muscle function, reduce pain and optimise loading of the trunk during full body movement.

4. In the identification of a psychogenic causes of back pain, supervised exercise could be tested to minimize kinesiophobia. This can often be complemented with patient education that can help pain management and enable self-care.

5. In the identification of a postural impairment, posture correction and ergonomic interventions can be tested.

Dosage of treatment measures should be individualised and sufficient to achieve the desired effect. Initial targeted treatment should be through individual patient care. As a complement to the initial targeted treatments, the

©Linköping University 01042017

purpose of a general training and patient education is to restore or improve function and activity. The suitability of group-based patient care is assessed in consultation with the patient as general training and patient education is considered relevant to support the patient's self-care.

©Linköping University 01042017

Clinical process and reasoning pathway - BetterBack☺

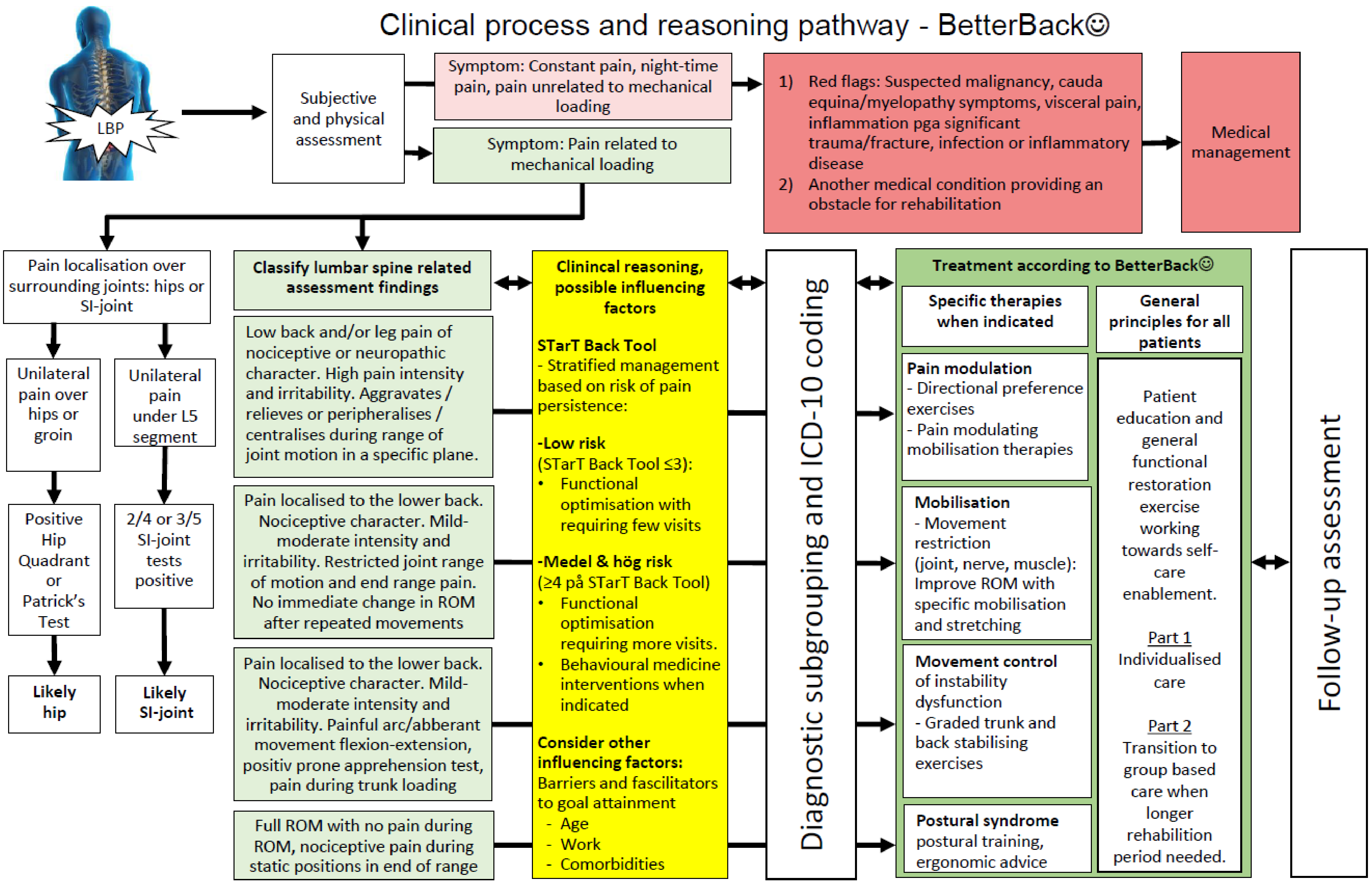

©Linköping University 01042017

6. BetterBack☺ Model part 1 – Patient education brochure

BetterBack☺

Information on Low Back Pain

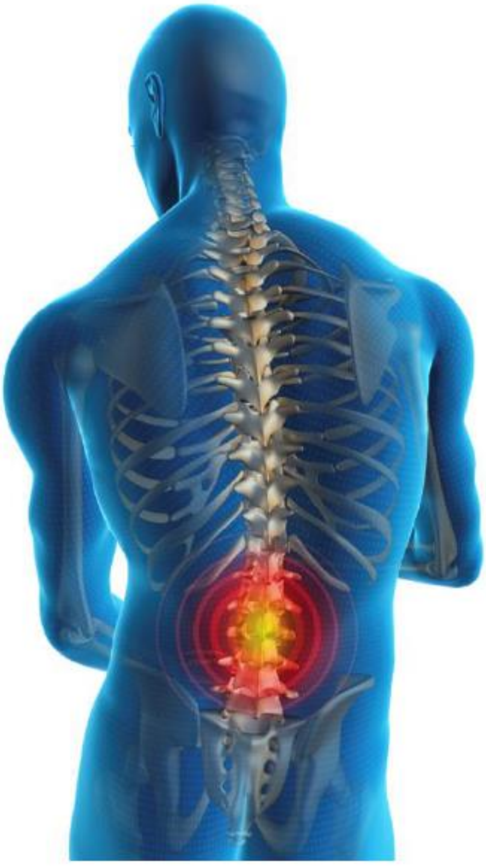

LINKÖPINGS UNIVERSITET

Region Östergötland

© Linköping University 20/03/2017

Low Back Pain

Low back pain (LBP) is a common harmless condition that affects almost everyone at some point. Over a one-year period, 4 out of 10 adults experience LBP. It is often characterised by varying degrees of pain and discomfort that may impact on ability to perform activities. An episode of LBP usually improves within 2-6 weeks. Most have a fairly stable pattern of back health for many years, which may sometimes be interrupted by a period of LBP. This is a normal pattern and does not mean that the condition is getting worse over time.

Degenerative changes in the spine

Something that astonishes many is that there is no direct connection between degenerative changes in the spine and common LBP. This means that changes seen on X-rays, magnetic cameras and computer tomography can show pronounced age related changes or disc herniation in a completely painless person, while someone with LBP may have very little or no changes.

The structure and function of the lower back and common causes of LBP

The lower back consists of many structures such as bones, joints, discs, stabilising ligaments, nerves, as well as deep and superficial muscles. Pain sensations may potentially be signalled by one or more structures of the lower back. It is often difficult to specify exactly if and which structures signal pain sensations. How we maintain an upright position in different situations is called posture. An optimal posture means that the spine has the best conditions for good mobility with optimal distribution of body weight. Suboptimal posture, suboptimal loading of the back or even too little loading of the back can be possible contributing factors of LBP.

Experience of back pain

Pain is first experienced when interpreted in the brain. How the pain is interpreted depends on experience, thoughts, feelings and expectations. In some cases, pain may be experienced in the lower back but in the absence of pain signals from structures in the lower back. The pain system may also become hypersensitive and in some cases the pain can persist even though the original cause of the pain has resolved.

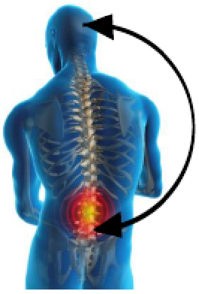

Figure 1. Pain is interpreted in the brain. This can be in the presence or absence of signals form lower back structures

© Linköping University 20/03/2017

©Linköping University 01042017

Back pain symptoms

In addition to back pain, you may have pain in the buttocks and in one or both legs. You may have difficulty standing, sitting, walking, bending etc. This can lead to frustration, depressed mood and anxiety. Some may be afraid of physical activity and become inactive. All of this can impact negatively on you everyday life.

Tips when you have a particularly troublesome period

Think about what you have read in this brochure, that pain comes in periods but usually calms down. Also think about what relieves the symptoms and what you can do when you have a troublesome period. You may have a favorite exercise or other strategy to manage troublesome periods. Contact your physiotherapist for help if you feel after 2-6 weeks that pain doesn't subside. If you have numbness and tingling in both legs, loss of skin sensation or weak muscles in the legs and feet and especially if you have trouble controlling your bladder and bowel you should seek medical care. If you have LBP after an accident or have been previously treated for cancer or osteoporosis, it is also important to seek medical care. For the vast majority, however, back pain is a harmless and common condition that comes and goes.

Back Health

Good back health is a balance between the back's capacity on one side of the scale and physical / mental stresses on the other side as in the figure below.

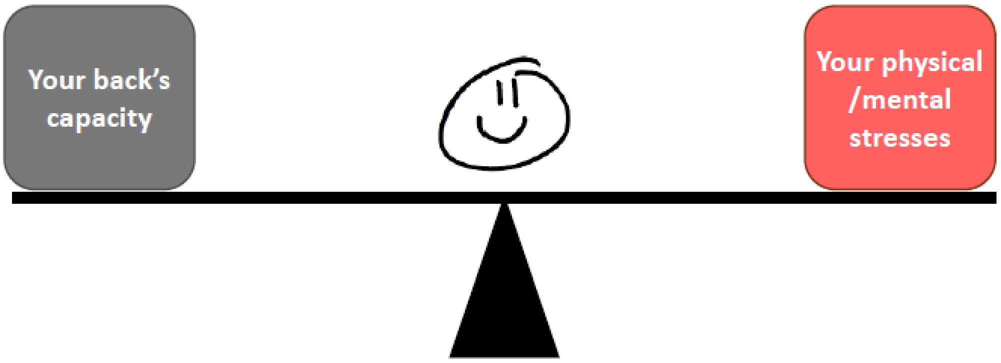

Figure 2. Balance between back capacity and stresses

© Linköping University 20/03/2017

©Linköping University 01042017

Back pain occurs when imbalance occures between back capacity compared to physical / mental stresses as in the figure below.

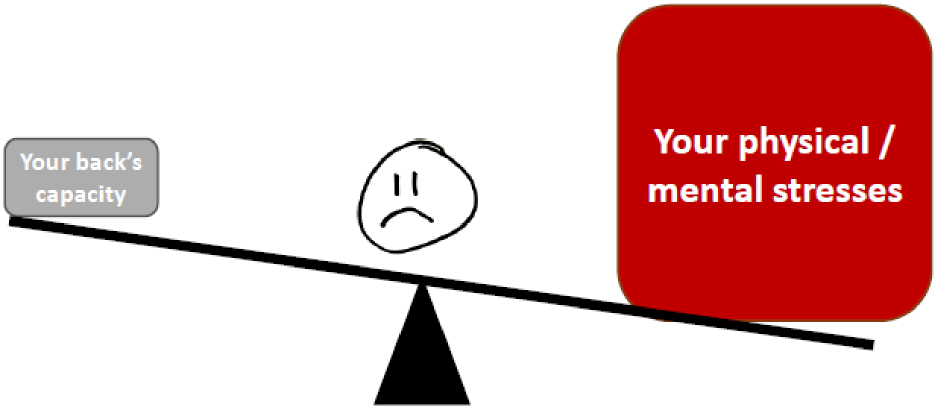

Figure 3. Imbalance between back capacity compared to physical / mental stresses

<u>General advice / self-care</u>

During the acute phase, most people are in need to take it easy and adjust their physical and mental stresses. Today, however, there is extensive research that recommends avoiding bedrest and instead modifying physical activity and successively returning to normal activities as quickly as possible. You can use a pain management scale to find the right level of back physical and mental stresses during everyday activities and also when you work out. This model is based on keeping you within acceptable perceived pain levels during an activity and within 24 hours after activity. This means that activity may increase the pain within acceptable pain levels during or after training, but it should return to initial levels within 24 hours. If you are unsure about the right level of back physical and mental stresses consult your physiotherapist.

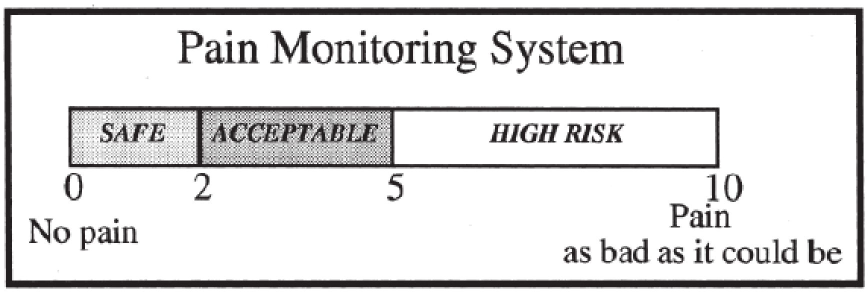

Bild 4. During activity, it is preferable that the pain is within safe to acceptable levels and that the pain returns to initial levels within 24 hours

© Linköping University 20/03/2017

©Linköping University 01042017

Treatment for back pain

The goal is to increase your back's capacity and reduce your physical and mental stresses. You can increase your back's capacity by optimising your back posture, muscle stength, muscle endurance, agility, and improving your overall fitness. You can reduce your physical and mental stresses by optimising your back's physical loads, reducing negative emotions through a positive approach and reducing everyday stress and changing your thoughts about your LBP

- Increase strength, endurance and agility
- Optimise posture
- Improve general fitness

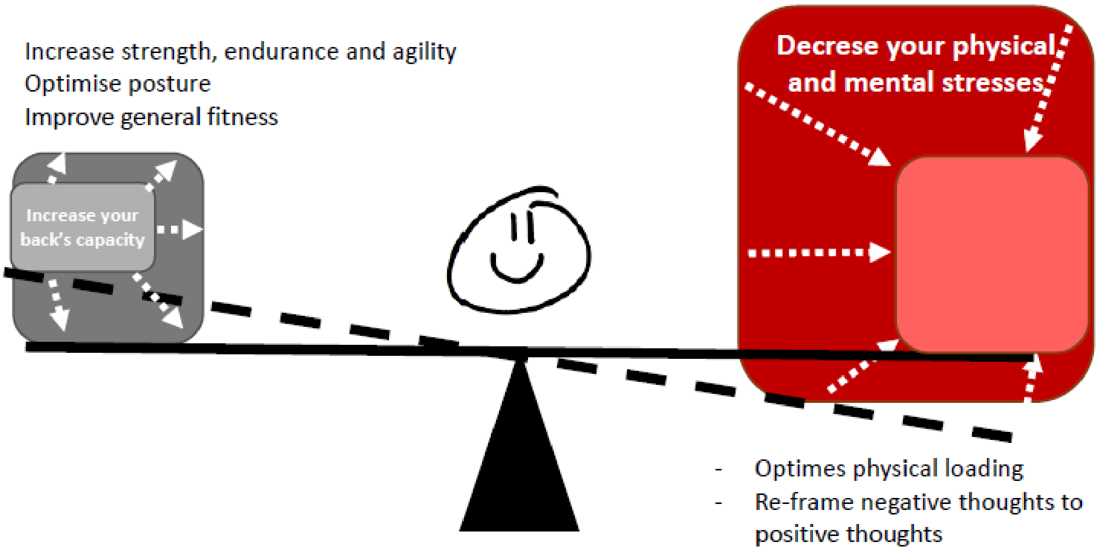

- Optimes physical loading
- Re-frame negative thoughts to positive thoughts
- Reduce emotional stress

Figure 5. How to balance the back's capacity and stresses

The BetterBack☺ model of care

The BetterBack☺ model of care for LBP focuses on evidence based physiotherapy, patient education and exercise. The main aim is to manage LBP symptoms and enable the patient's self-care ability. You will receive a thorough assessment and individualised care. Depending on your need for extended support in addition to your physiotherapist's initial interventions, pain education seminars and supervised exercise in a group format can be provided for 6 weeks, 2 times / week. The pain education seminars include explanatory models of what pain is, different ways of managing pain, as well as how to balance your back capacity and your physical and mental stresses you are exposed to. It is common for people to become less physically active after a troublesome period of LBP. It is therefore important to get started with some form of general fitness training. You can improve general fitness by walking, Nordic walking, cycling, jogging and swimming. If you experience pain during activity, you can use the pain management scale (see Figure 4). It is important that you feel motivated and can adapt your training to fit into your everyday life. In the BetterBack☺ model of care program, you can get help on how to get started!

© Linköping University 20/03/2017

©Linköping University 01042017

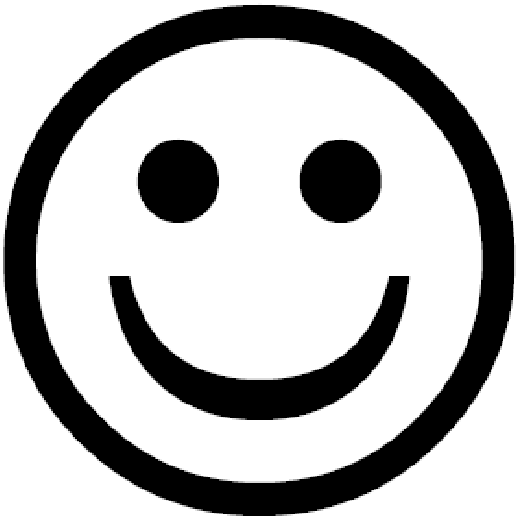

© Linköping University 20/03/2017

©Linköping University 01042017

7. BetterBack☺ Model part 2 – Group education seminar for patients

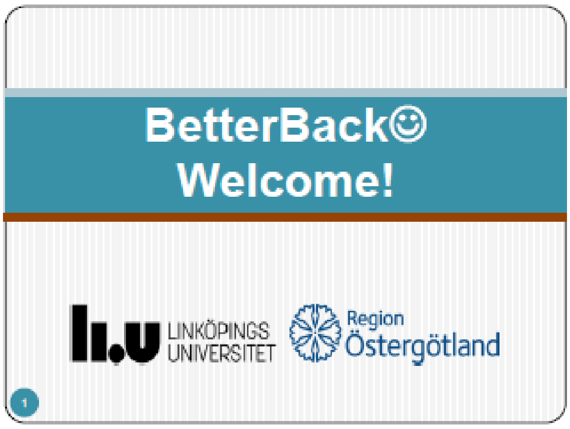

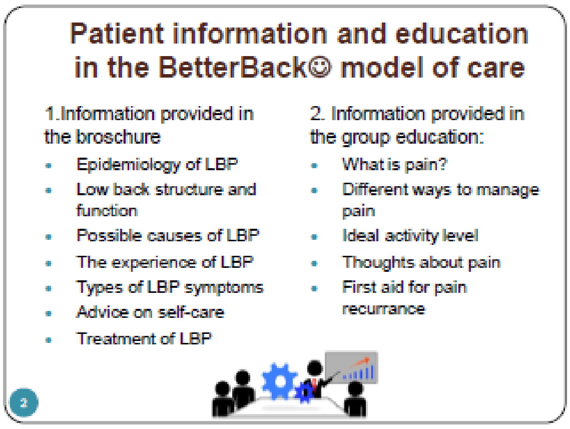

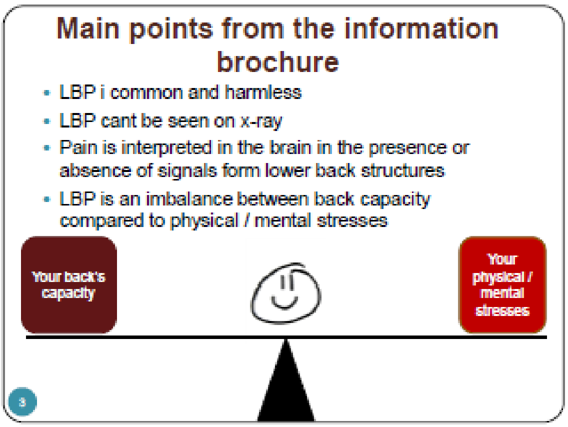

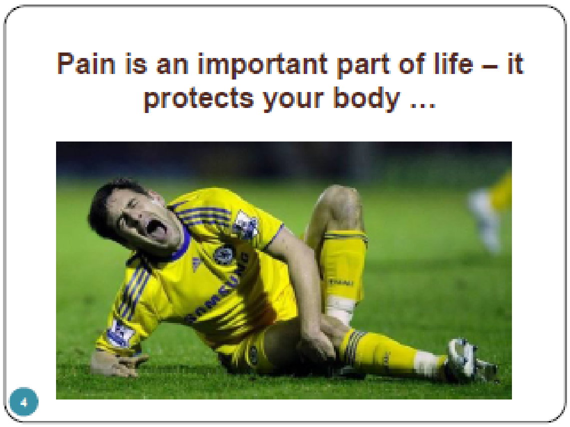

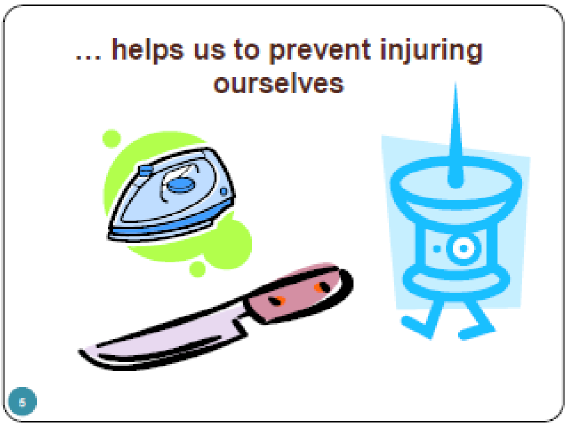

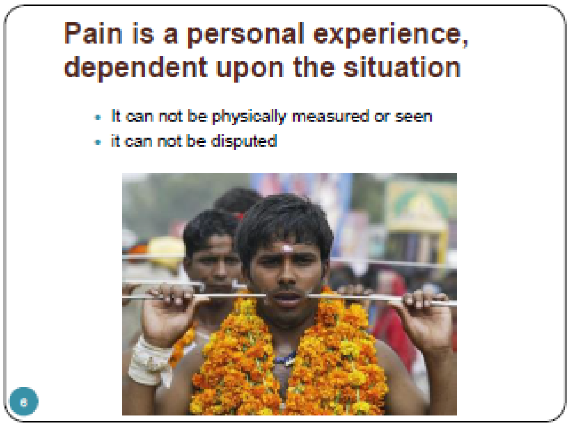

©Linköping University 01042017

The situation affects the pain experience

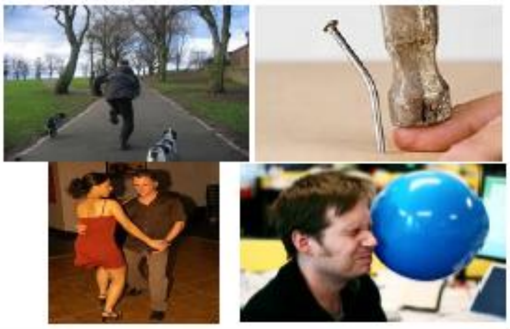

7

Pain experience =
warning from the body – interpretation in the brain

The body

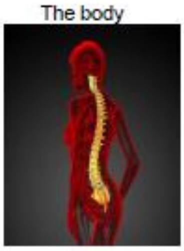

+ situation + thoughts + experience

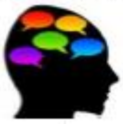

Pain is inhibited or strengthened

↓

Pain experience

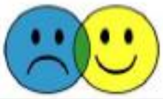

8

The body's own pain relief system

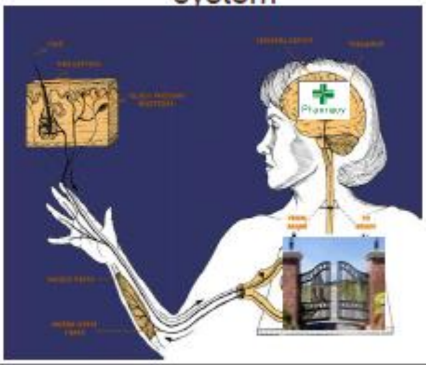

9

Pain experience

- Pain can be **dampened**
  - If you understand it's cause
  - If it is predictable
  - If you know how to handle the pain
  - If it is manageable
  - If you have a positive attitude
  - If you are goal-focused

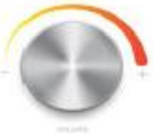

10

Pain experience

- Pain can be **dampened**
  - If you understand it's cause
  - If it is predictable
  - If you know how to handle the pain
  - If it is manageable
  - If you have a positive attitude
  - If you are goal-focused
- Pain can be **aggravated**
  - If you feel uncertain of its cause
  - If it is unpredictable
  - If you cant control the pain
  - If you have a depressed mood
  - If you generally feel pressured
  - If it is associated with bad experiences

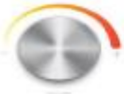

- What dampens / aggravates your pain?

11

Thoughts affect our self-perception…,

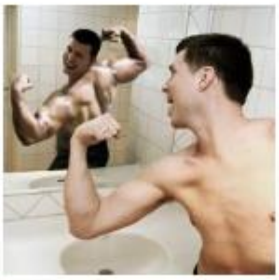

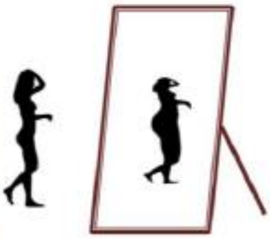

12

©Linköping University 01042017

… and thoughts affect the pain experience

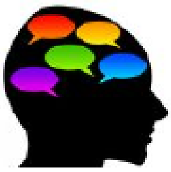

You are going to be immunized against influenza. You know the needle hurts for a few days. How do you experience the pain?

You have back pain after a longer walk. How do you experience pain?

13

… and thoughts affect the pain experience

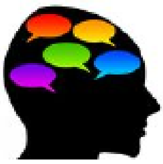

HEY MACARENA!

- **Prolonged back pain can be like a bad song that gets stuck in your mind. The brain has learned it without you wanting to and it plays over and over again .... The more irritating the song the more easily it gets stuck in your mind**

You started training but get more back pain afterwards. It may be muscle soreness or it may be the playing over and over again of pain memories like "the known song". How do you relate to this?

14

Pause exercise

Try to breathe relaxed and calm, focus on breathing for 2 minutes

Acknowledge your pains in a neutral way, but keep the focus on breathing

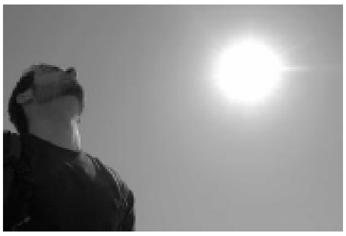

15

Pause Exercise -redirecting

What happens to your pain experience when you focus on your breathing?

Does this redirect your thoughts away from attention to pain?

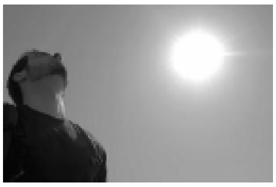

16

Pain coping

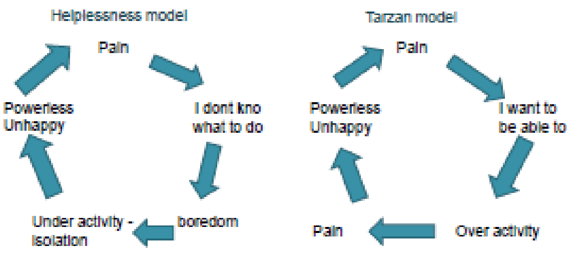

Different strategies. "Helplessness" or "Tarzan model"?
Are there other strategies? In which way do you react?
What consequences does this have?
Advantages/disadvantages?

17

Activity level – too much activity Tarzan model

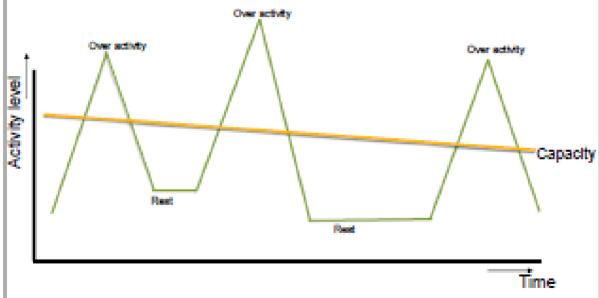

18

©Linköping University 01042017

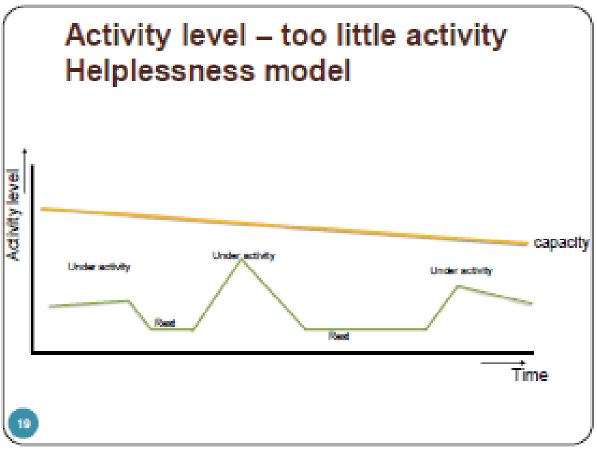

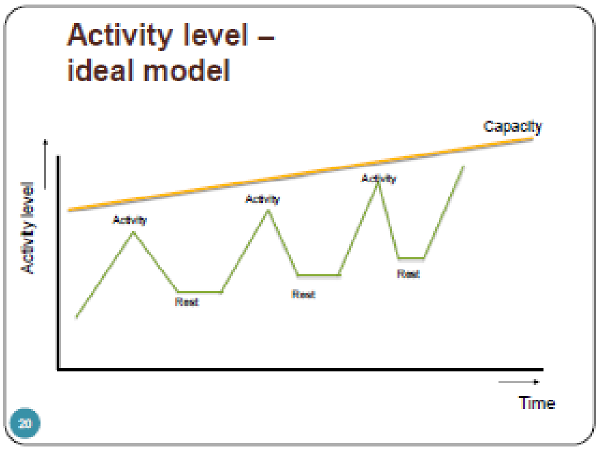

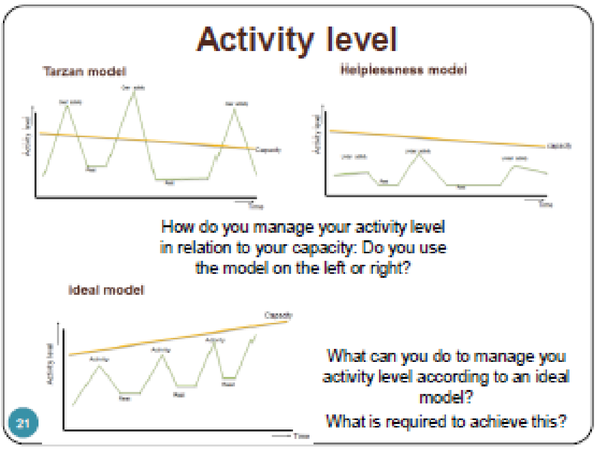

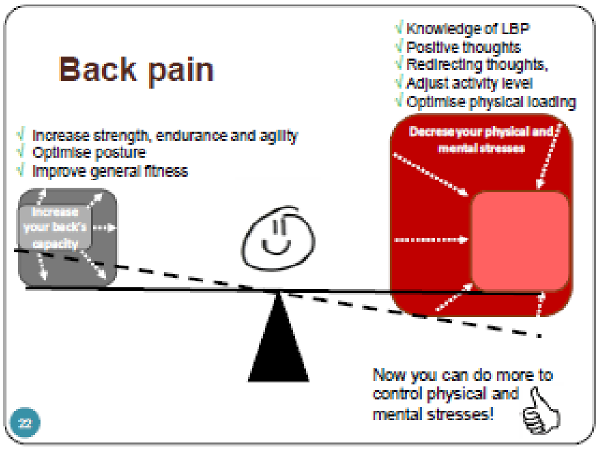

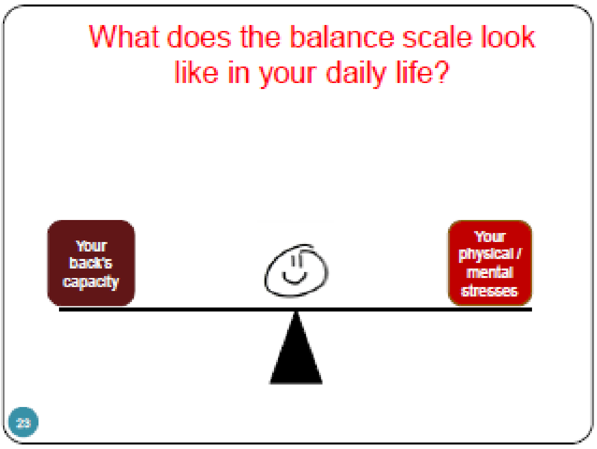

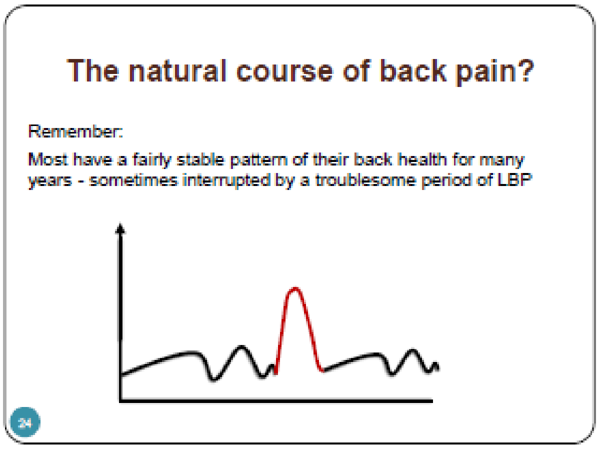

©Linköping University 01042017

First aid when back pain flares up

- How long time do you expect the pain to be aggravated?
- What can you do when the pain gets worse?
- Do you have a favorite strategy to reduce pain?
- What can you do to make it easier for yourself?
- Ask for help?

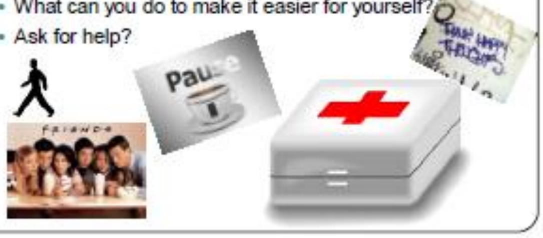

25

Tips…

- Adjust activity and load according to your back capacity, not too much not too little
- Distribute activity throughout the day
- Be active, take short breaks
- Think positive thoughts
- Help youself
- And ask for help from others

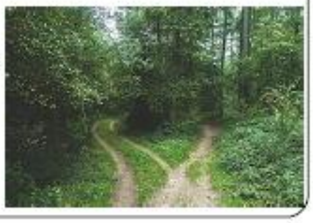

26

Back pain

√ Knowledge of LBP
√ Positive thoughts
√ Redirecting thoughts,
√ Adjust activity level
√ Optimise physical loading

√ Increase strength, endurance and agility
√ Optimise posture
√ Improve general fitness

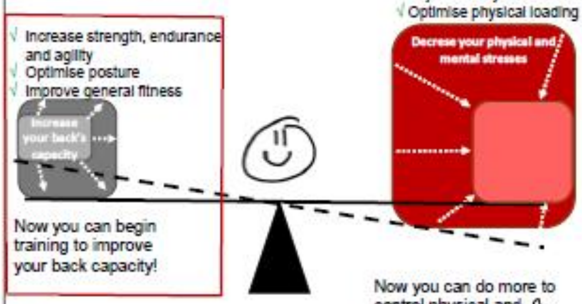

Now you can begin training to improve your back capacity!

Now you can do more to control physical and mental stresses!

22

Increase your capacity – improve general fitness

Help yourself to be physically active to optimising brain and body's wellbeing.

- Improved memory & concentration
- Better coping with stress
- Improved mood
- Divert negative thoughts
- Contributes to better health, physical and mental

Physical activity during acute LBP?

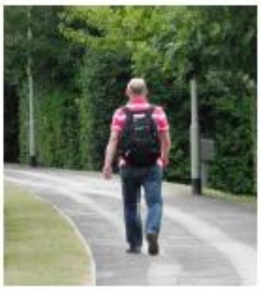

28

Reduce sedentary behaviour

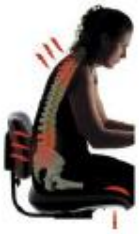

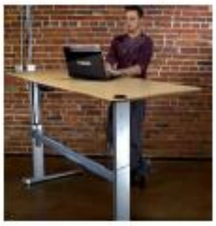

How can i change too little exercise and too much sitting ?

29

Increase your back's capacity! We will help you get started!

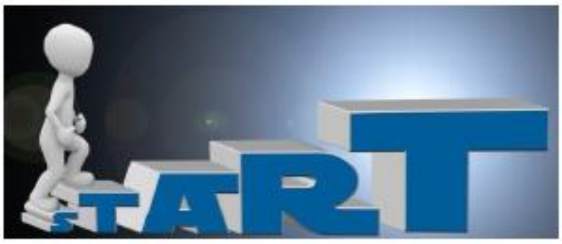

30

©Linköping University 01042017

Training - BetterBack☺

- Supervised exercise 2 times / week for 6 weeks, then self-mediated for 6 weeks
- A little bit of training is better than none
- Remember that training can give temporary muscle soreness which is not a worsening of back pain
- Your back is not fragile, the "well known pain memory song" can activate also during exercise
- Talk with your physiotherapist about a long term plan after BättreRygg☺

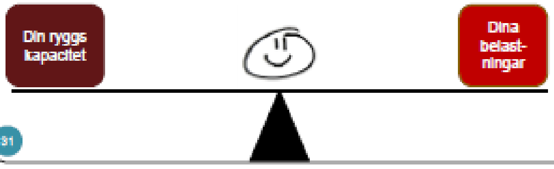

31

Summary

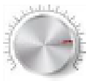

- Pain can be aggravated or dampened by many factors

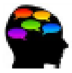

- Thoughts affect the pain experience

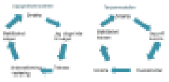

- There are different ways of coping with pain

32

Summary

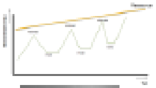

- You can use your capacity optimally

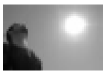

- You can redirect your thoughts

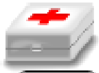

- If your back pain gets worse, do you have a plan!

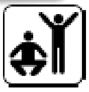

- Training increases your back's capacity!

33

©Linköping University 01042017

8. BetterBack☺ Model – Training program for patients

Training program for patients receiving the BetterBack☺ model of care for LBP		
Part 1: Posture, muscle control and coordination of basic body movements	Goal: To ensure the patient has satisfactory posture and trunk muscle activation in static positions as well as in conjunction with basic body movement in the sitting, sitting and standing.<br>Implementation*: Exercises and dosages are individually adjusted by the treating therapist. Exercises are performed as home programs and daily training is recommended for optimal results.<br><br>*The therapist assesses when basic competencies in program 1 are achieved before progressing to program 2*.	Training range of movement<br>Goal: Restore normal mobility.<br><br>Implementation: Individualise based on if the patient has movement restriction.
Part 2: Graded training of muscle strength, coordination and endurance	Goal: To ensure the patient has satisfactory ability to perform more challenging body movements with adequate strength, corrdination and endurance.<br>Implementation*: Exercises and dosages are individually adjusted by the treating therapist. Exercises are performed twice a week for 12 weeks with follow-up conducted by the treating therapist. During the first 6 weeks, patients are offered the opportunity to train in a group supervised by a physiotherapist. The patient will then receive support and feedback regarding the practice of exercises and help to upgrade exercises if necessary. Patient education on self-care and management of back pain is also performed in groups.	
***Prerequisite for upgrading the training program is that the patient can satisfactorily perform basic exercises for posture and trunk control in Part 1. Using Part 2 as a basis, the physiotherapist selects and individualises relevant exercises and dosing based on the assessment findings. If support with the traning program is required (in addition to a self-mediated home based program), group traning supervised by another therapist can implemented. However, the follow-up of the patient is still the responsibility of the therapist who first assessed and initiated the patient's treatment plan. The program is designed with graded levels where difficulty level is increased by successively progressing from stages A through to C. Patients are to perform the exercises as instructed. Training can initially produce some muscle soreness, but this is normal and decreases gradually. Contact your physiotherapist if you have questions or feel unsure.**		

©Linköping University 01042017

Part 1. Posture, muscle control and coordination of basic body movements

1a. Basic trunk muscle activation and control in a lying position

Pelvic control exercise

- Lay on your back with your knees bent. Put your hands under your pelvis. Press your lower back down so it flattens down on the surface you are laying on. Feel how the pelvis tilts backwards and has rolled over your hands. Tip the pelvis forward and feel how the lower back rises again. Remove your hands and repeat the tipping forward and backward with less and less movement. Stop when you come to a normal neutral pelvic position.

Activating your inner trunk muscles

This exercise focuses on the activation of core muscles in your back, abdomen and pelvis. It is also known as ”<u>core activation”</u>

- Lay on your back with your knees bent and put your hands on your waist.
- ① Breathe calmly in and out and make an ssss sound and feel your fingers how the inner muscles between your pelvis bones become activated. This muscle activation should be done slowly and with a minimal force where you feel that the lower part of the stomach is pulled inward-backward-upward.
    - Alternative instructions
        - Draw the lower part of your stomach inwards from the waist of you pants
        - Imagine that you activate your lower stomach muscles just like if you were tightening av belt around you waist
        - Imagine that your holding on to go to the toalet
- <u>Make sure that **you dont**</u>:
    - Hold your breath, press your lower back down or bend your back forward

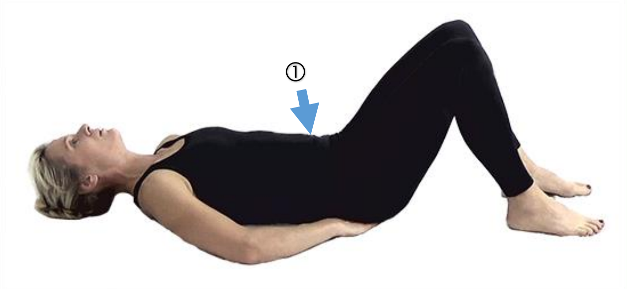

1b. Basic trunk muscle activation and control in conjunction with body movement in a lying position

In conjunction with leg movement

Lay on your back with your knees bent. ① Start with ”core activation” ② Move your knee on one side out towards the side with and back to the middle with slow controlled movement. Repeat alternately on each side. Maintain a stable positioning of your trunk and pelvis.

Repetitions________________

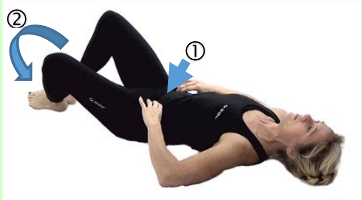

Perform the same exercise in side lying with movement of one leg. Perform even on the other side thereafter

Repetitions________________

In conjunction with arm movement

① Start ”core activation”. ② Bring your arms up över your head, together or alternately, with slow controlled movement. Maintain a stable positioning of your trunk and pelvis.

Repetitions_________________

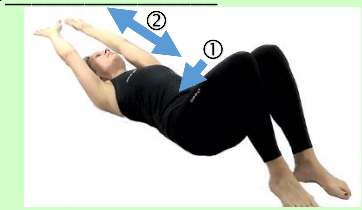

©Linköping University 01042017

2a. Basic trunk postural control in a sitting position

With neutral posture, loading of the spine is optimally distributed. Feel how the physical loading on your back increases when you sit with hunched posture, and how it relieves when you hold a neutral posture.

Training of posture in sitting position:

- Sit on a chair with your hands under your buttocks.
- ① Rotate your pelvis forward over your hands. You should feel like you are arching your back more. Rock your pelvis backward so you return to a neutral back posture. ② Rotate your pelvis backwards so that you have a hunched posture. Continue to rotate your pelvis backards and forwards a few times

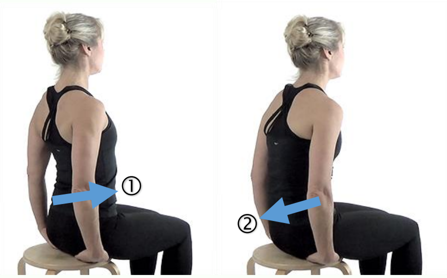

- Stop in a position where you feel you have a even weight distribution over your hands and neutral back posture.
- Your ears, shoulders and hips should create a straight line vertically.

2b. Basic trunk muscle activation in a sitting position

Sit on a chair with good posture. ① Train holding a ”core activation”.

Repititions________________

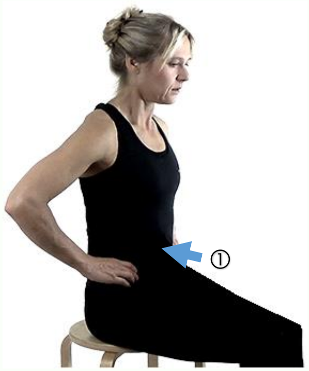

2c. Basic trunk muscle activation and control in conjunction with body movement in a sitting position

In conjunction with leg movement

Sit on a chair or training ball. ① Start with ”core activation”. ② Lift up your knees alternately with slow controlled movement. Maintain a stable positioning of your trunk and pelvis.

Repetitions________________

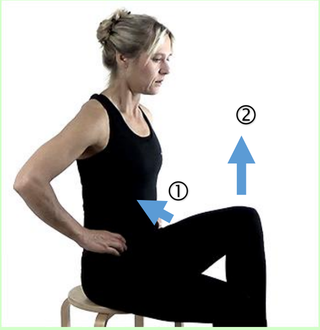

In conjunction with arm movement

① Start ”core activation”. ② Bring your arms up över your head, together or alternately, with slow controlled movement. Maintain a stable positioning of your trunk and pelvis.

Repetitions_________________

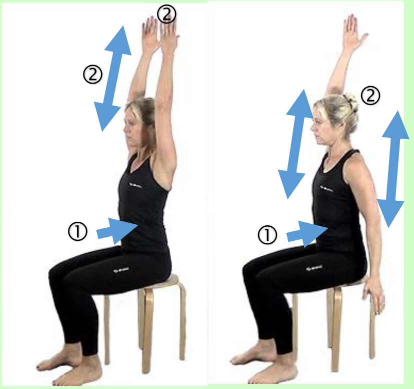

©Linköping University 01042017

3a. Basic trunk postural control in a standing position

With neutral posture, loading of the spine is optimally distributed. Feel how the physical loading on your back increases when you sit with hunched posture, and how it relieves when you hold a neutral posture.

Training of posture in sitting position:

- Stand with your feet hip width apart
- ① Shift your weight forwards and backwards and find a neutral weight distribution over the soles of your feet.
- ② Bend and straighten your knees a few times and find the position where your knees are slightly bent.
- ③ Tilt your pelvis forwards and backwards a few times and the position in the middle where you pelvis has a neutral position.
- ④ Move your head backwards with your chin in.
- ⑤ Bring your shoulders up and then relax your shoulders.
- Your ears, shoulders, hips, knees and feet should now be in a straight line.

① ② ③ ④ ⑤

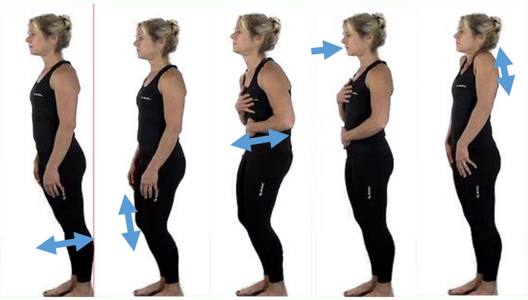

3b. Basic trunk muscle activation in a standing position

Stand with a neutral posture. ① Train holding a ”core activation”.

Antal________________

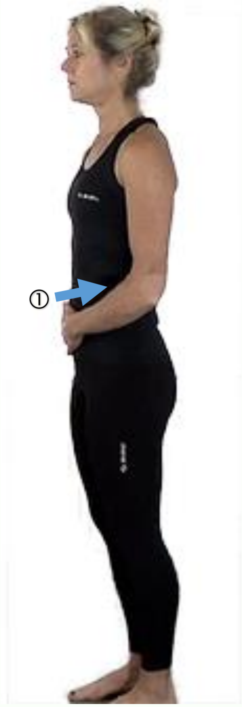

3c. Basic trunk muscle activation and control in conjunction with body movement in a standing position.

In conjunction with weight transfering

Stand with a neutral posture. Place you feet wide apart. ① Start ”core activation”. ② Transfer your weight from one leg to the other alternately. Maintain a stable positioning of your trunk and pelvis.

Repetitions________________

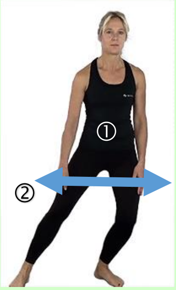

In conjunction with arm movement

Stand with a neutral posture. ①Start ”core activation”. ② Bring your arms up över your head, together or alternately, with slow controlled movement. Maintain a stable positioning of your trunk and pelvis.

Repetitions_________________

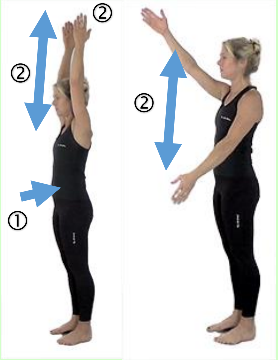

©Linköping University 01042017

Part 2: Graded training of muscle strength, coordination and endurance		
**Difficulty level A**	**Difficulty level B**	**Difficulty level C**
**1A) Pelvis lifts in lying position**<br>Lay on your back with your knees bent and arms by your side.<br>① Start with ”core activation”.<br>② Lift up your pelvis from the floor.<br>Repetitions________________<br>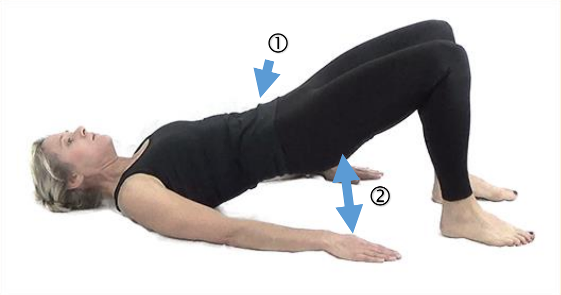<br>Tip: Increase resistance by using theraband placed over you pelvis and hold the ends down with your hands.<br>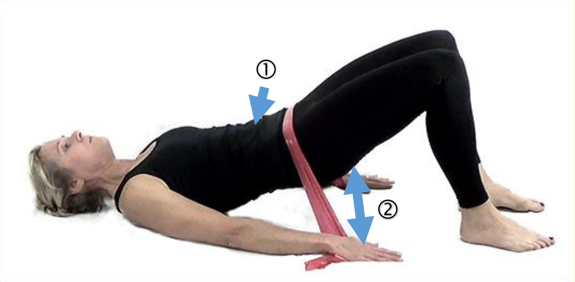	**1B) Pelvis lifts + leg kicks in lying position**<br>Lay on your back with your knees bent and arms by your side.<br>① Start with ”core activation”.<br>② Lift up your pelvis from the floor.<br>③ Lift and extend one leg while maintaining a stable positioning of your trunk and pelvis. Lower your foot to the floor again and lower the pelvis. Repeat and change legs every time.<br>Repetitions________________ each side<br>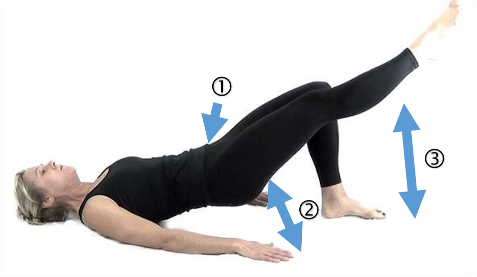<br>Tip: Increase resistance by using theraband placed over you pelvis and hold the ends down with your hands.	**1C) Single leg pelvis lift i lying position**<br>Lay on your back with your knees bent and arms by your side.<br>① Start with ”core activation”.<br>② Lift up your pelvis from the floor and at the same time lift and extend one leg. Lower your foot to the floor again and lower the pelvis. Repeat and change legs every time.<br>Repetitions________________ each side<br>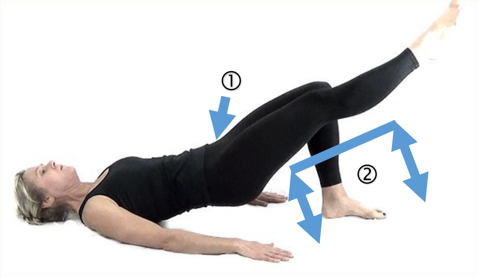<br>Tip: Increase resistance by using theraband placed over you pelvis and hold the ends down with your hands.

©Linköping University 01042017

2A) Knee lifts in lying position

Lay on your back with your knees bent and put your hands on your waist.

① Start with ”core activation”.

② Lift one fot slowly up by bending your hip while maintaining a stable positioning of your trunk and pelvis. Slowly bring your fot back to the floor. Repeat and change legs every time.

Repetitions________________ each side

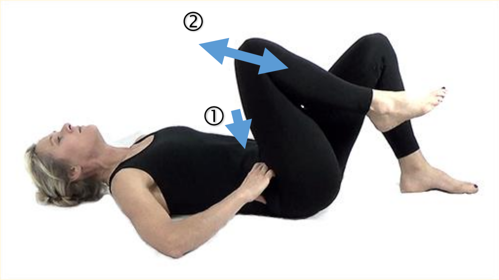

2B) Straight leg raises in lying position

Lay on your back with your knees bent and put your hands on your waist.

① Start with ”core activation”.

② Extend and lift one leg while maintaining a stable positioning of your trunk and pelvis. Slowly bring your leg back to the floor. Repeat and change legs every time.

Repetitions________________ each side

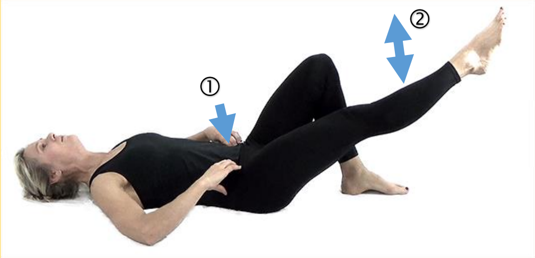

2C) Rotating sit-ups in lying position

Lay on your back with your knees bent.

① Start with ”core activation”.

② Place your hands behind your head and bring your opposite knee and elbow together by bending you back forwards. Repeat alternately on each side.

Repetitions________________ each side

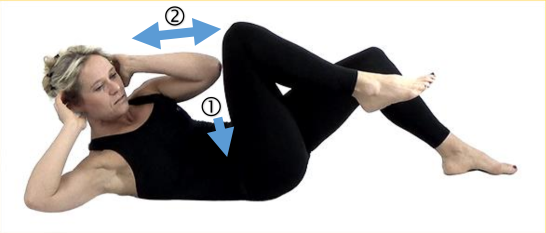

©Linköping University 01042017

3A) Hip muscle training in lying position
Lay on your back with your knees bent and arms by your side. Tie a theraband around your knees.
① Start with ”core activation”.
② Move your knees slowly away from each other and slowly back again while maintaining a stable positioning of your trunk and pelvis.

Repetitions________________

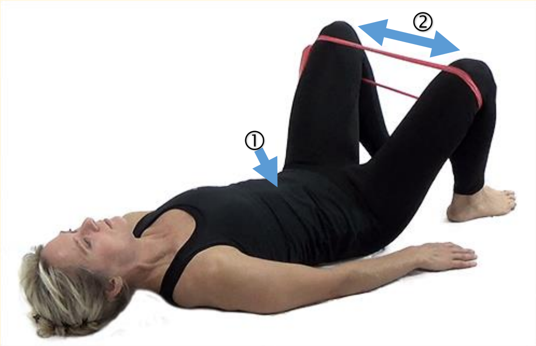

3B) Hip muscle training in side lying position
Lay on your side with your knees bent. Tie a theraband around your knees.
① Start with ”core activation”.
② Move your top knee slowly away from the other and slowly back down again while maintaining a stable positioning of your trunk and pelvis.

Repetitions________________ each side

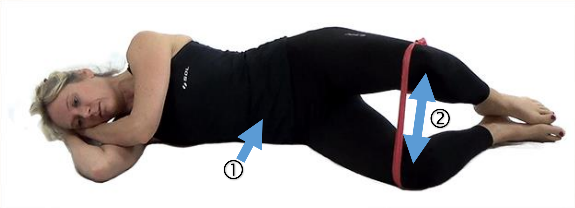

3C) Hip muscle training in side lying position
Lay on your side with your legs straignt. Tie a theraband around your ankles.
① Start with ”core activation”.
② Move your top leg slowly away from the other and slowly back down again while maintaining a stable positioning of your trunk and pelvis.

Repetitions________________ each side

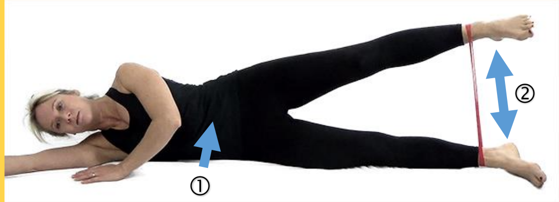

Alternative
Stand on one leg in a crouched position. Straighten up and move your free leg diagonally backwards just like skating. Repeat alternately on each side.

©Linköping University 01042017

4A) Side plank + arm movement

Lay on your side with support of your lower arm and knee and lift up your pelvis.

① Start with ”core activation”.

② Maintain a stable positioning of your trunk and pelvis while bringing your free arm up over your head.

The exercise can be done with the pelvis still (static) or by moving the pelvis up and down (dynamically). Perform also on the other side.

Repetitions________________ each side

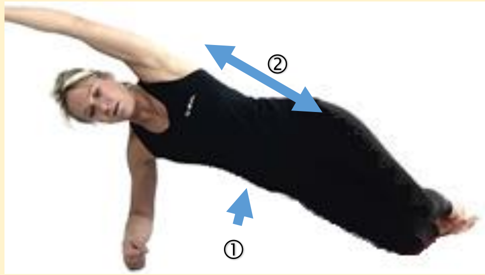

4B) Side plank + arm movement

Lay on your side with support of your lower arm and feet and lift up your pelvis.

① Start with ”core activation”.

② Maintain a stable positioning of your trunk and pelvis while bringing your free arm up over your head.

The exercise can be done with the pelvis still (static) or by moving the pelvis up and down (dynamically). Perform also on the other side.

Repetitions________________ each side

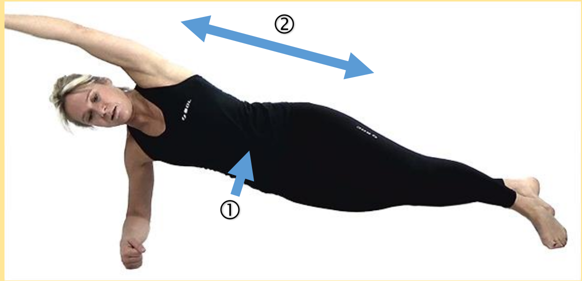

4C) Side plank + arm movement

Lay on your side with support of your lower arm and feet and lift up your pelvis.

① Start with ”core activation”.

② Maintain a stable positioning of your trunk and pelvis while bringing your free arm up and rotating your back.

Repetitions________________ each side

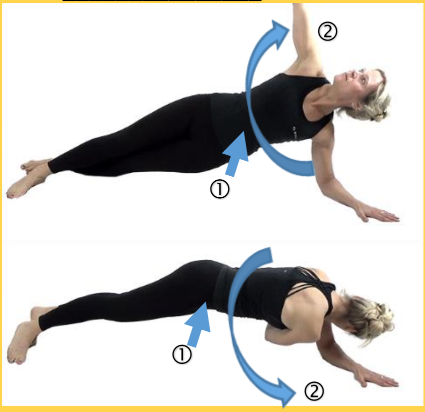

Alternative: Stand beside a therband tied to a pole. Pull the theraband diagonally across your body and rotate your back.

Repetitions________________ each side

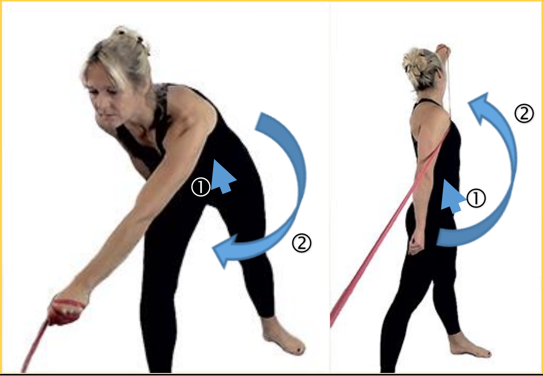

©Linköping University 01042017

5A) Chair plank

Stand on your knees and support your lower arms on a chair or pilates ball.

① Start with ”core activation”.

② Maintain a stable positioning of your trunk and pelvis while you lift your knees from the floor. Hold ________ seconds. Bring your knees back down to the floor.

Repetitions________________

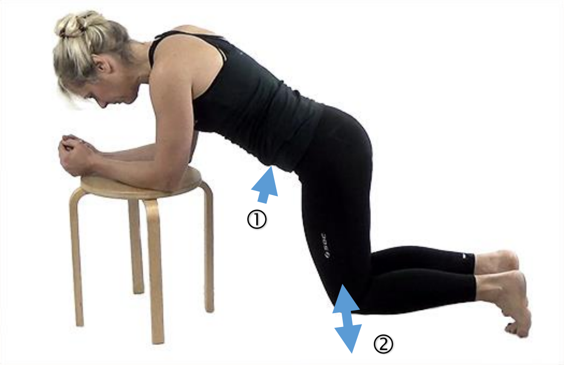

5B) Floor plank

Stand on your knees and support your lower arms on the floor.

① Start with ”core activation”.

② Maintain a stable positioning of your trunk and pelvis while you lift your knees from the floor. Hold ________ seconds. Bring your knees back down to the floor.

Repetitions________________

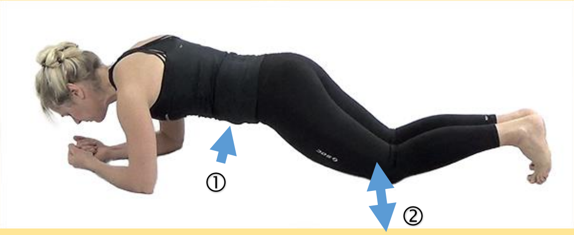

5C) The plank + leg lifts

Stand on your knees and support your lower arms on the floor.

① Start with ”core activation”.

② Maintain a stable positioning of your trunk and pelvis while you lift your knees from the floor holding your legs straight. Lift one foot up from the floor and hold ________ seconds. Bring your foot back down to the floor.

Repetitions________________ each side

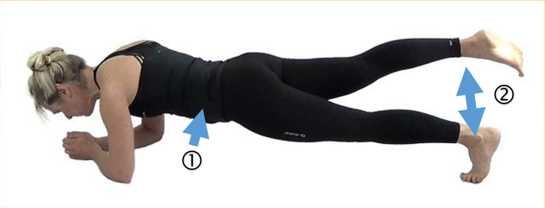

©Linköping University 01042017

6A) 4-point kneeling superman exercise

Position yourself on your hands and knees with your back straight.

① Start with ”core activation”.

② Maintain a stable positioning of your trunk and pelvis while you lift up and down one arm alternately. Try instead one leg alternately. When this is easily accomplished, combined these so that you lift an arm and opposite leg up and down simultaneously and alternate sides.

Repetitions________________ each side

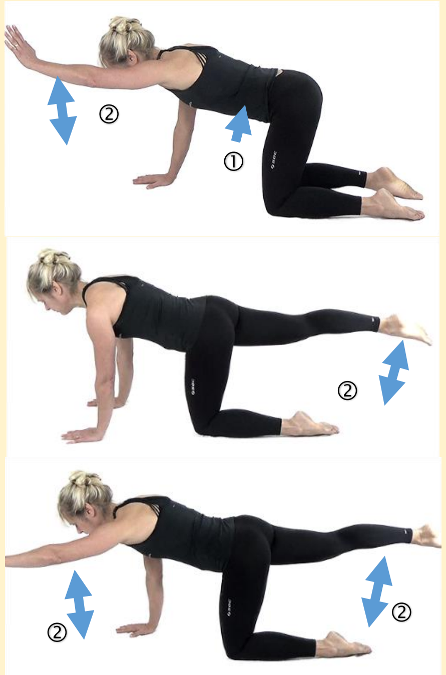

6B) 4-point kneeling theraband exercise

Posititionn yourself on your hands and knees with your back straight. Tie a theraband around your fot and hold on to the other end with your hands.

① Start with ”core activation”.

② Lift up and straighten your leg. Hold 5 seconds and then bring your leg down again.

Repetitions________________ each side

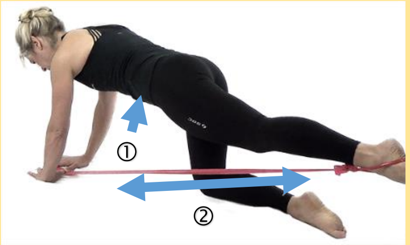

6C) Superman exercise with theraband

Position yourself on your hands and knees with your back straight. Tie a theraband around your fot and hold on to the other end with your opposite hand.

① Start with ”core activation”, curl your back and bring your opposite knee and elbow together while holding the theraband.

②. Slowly straighten your back, arm and opposite leg to stretch out the theraband. Perform the movement with good control of motion.

Repetitions________________ each side

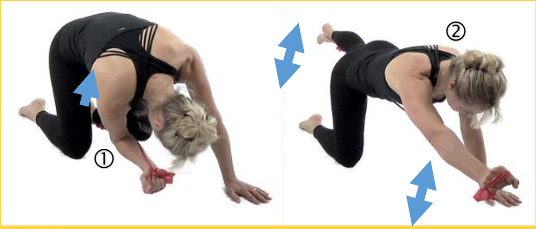

Alternativ: Try performing the same exercise while standing on one leg.

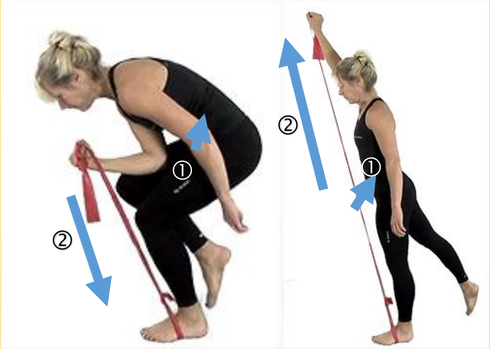

©Linköping University 01042017

7A) Push-ups against a wall

① Start with ”core activation”

② Perform push-ups against a wall while maintaining straight back posture.

Repetitions________________

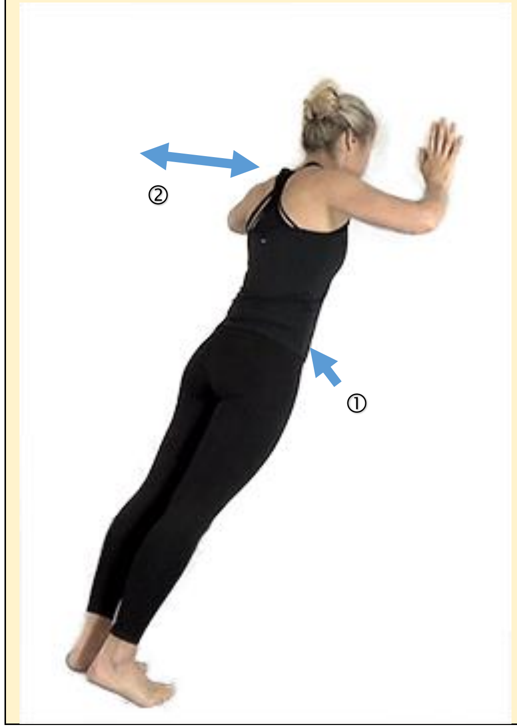

7B) Push-ups against a table

① Start with ”core activation”

② Perform push-ups against a table while maintaining straight back posture.

Repetitions________________

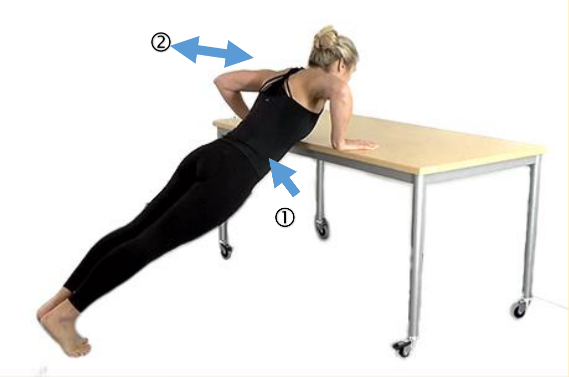

7C) Push-ups on the floor

① Start with ”core activation”

② Perform push-ups while maintaining straight back posture.

Repetitions________________

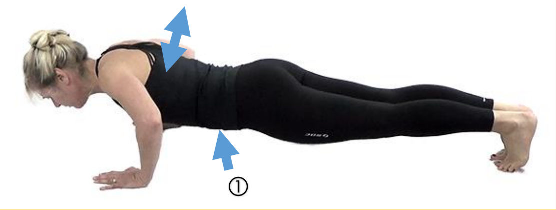

Alternativ: Try performing the same exercise with your feet on a pilates ball.

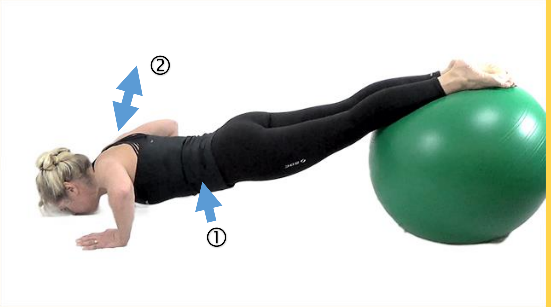

©Linköping University 01042017

8A) Standing arm lifts
Hold on to the ends of a theraband and stand on the middle of theraband
① Start with "core activation".
② Maintain a straight back posture while you lift your arms up over your head against the resistance of a theraband.

Repetitions________________

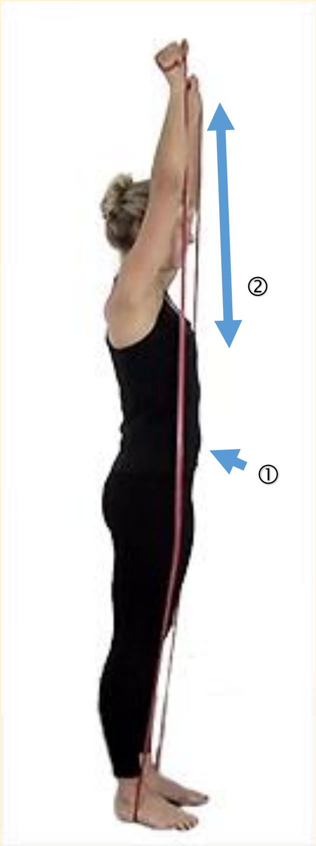

8B) Standing rows
Hold on to the ends of a theraband placed around a pole.
① Start with "core activation".
② Maintain a straight back posture while you perform arm rows alternately from side to side.

Repetitions________________

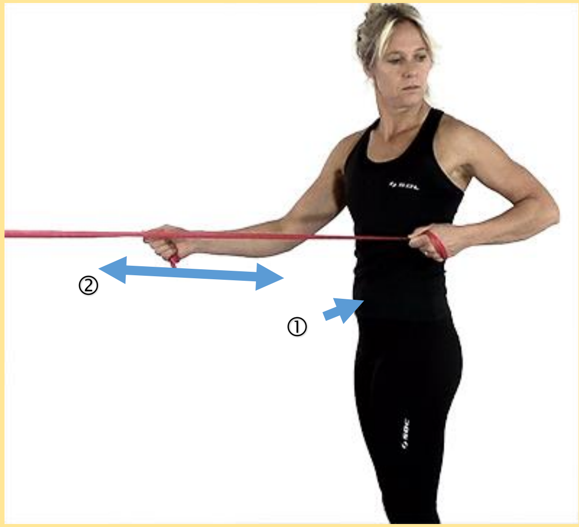

8C) Standing straight arm lifts
Hold on to the ends of a theraband and stand on the middle of theraband.
① Start with "core activation".
② Maintain a straight back posture and straight arms while you lift your arms alternately against the resistance of a theraband.

Repetitions________________ each side

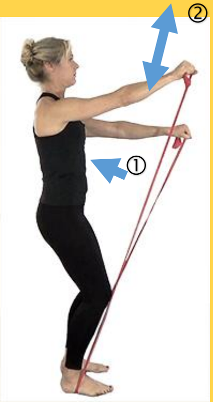

Alternative: Try performing straight arm ski rows.

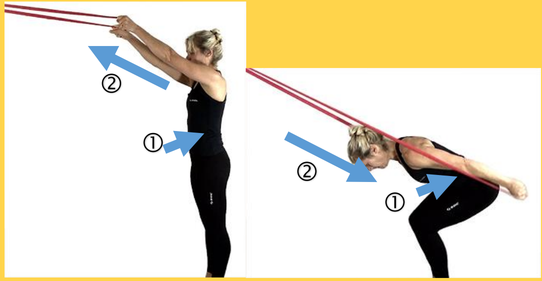

©Linköping University 01042017

9A) Squats

Stand with your back against the wall or with a pilates ball between your back and the wall. Place your feet hip width apart.

① Start with ”core activation”.

② Maintain a straight back posture while you perform a squat up to about 90 degrees of knee and hip bending.

Repetitions________________

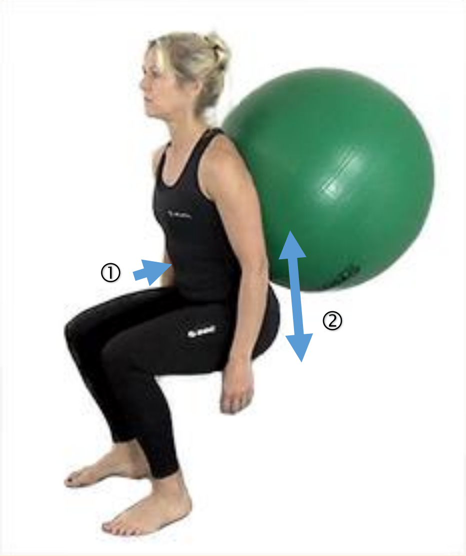

9B) Squats with your arms över your head

Stand with your back against the wall or with a pilates ball between your back and the wall. Place your feet hip width apart and your hands över your head.

① Start with ”core activation”.

② Maintain a straight back posture while you perform a squat up to about 90 degrees of knee and hip bending.

Repetitions________________

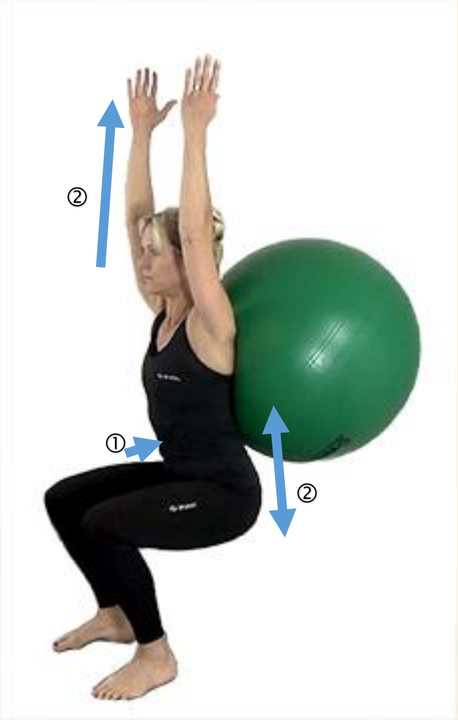

9C) Standing high knee lifts

Stand with your back against the wall, place your feet hip width apart and your arms on the wall.

① Start with ”core activation”.

② Maintain a straight back posture while you perform high knee lifts with alternating legs.

Repetitions________________ each side

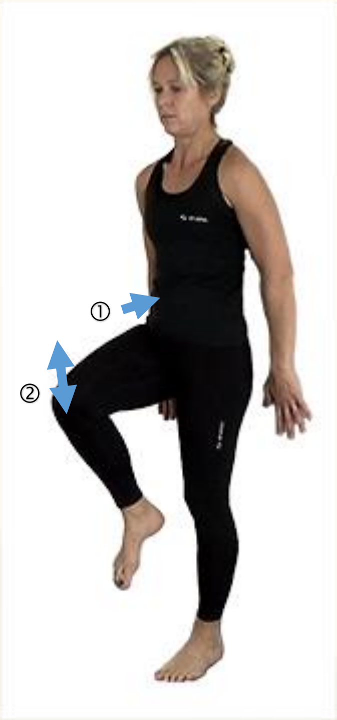

©Linköping University 01042017

10A) Tandem stance lunging weight tranfers

Stand with one foot a step length in front of the other foot.

① Start with ”core activation”.

② Maintain a straight back posture while you perform weight transfer forwards and backwards from foot to foot. Try even with your other foot forward.

Repetitions________________ each side

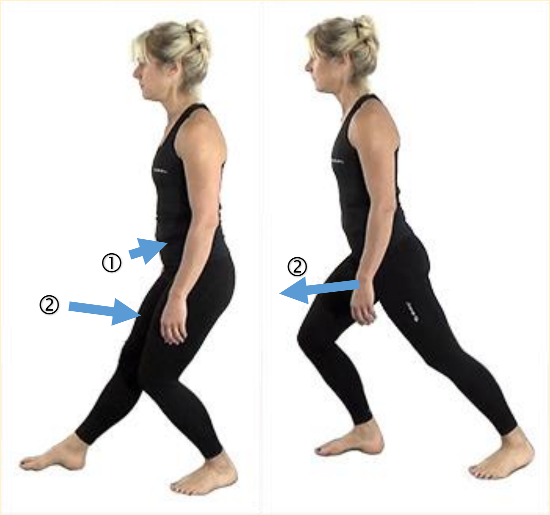

10B) Lunges

Stand with your feet hip width apart and your arms up horizontal to your body.

① Start with ”core activation”.

② Maintain a straight back posture while you perform forward lunges by taking av step forward with your weight over that leg och then taking a step back again. Alternate which foot you step forward with.

Repetitions________________ each side

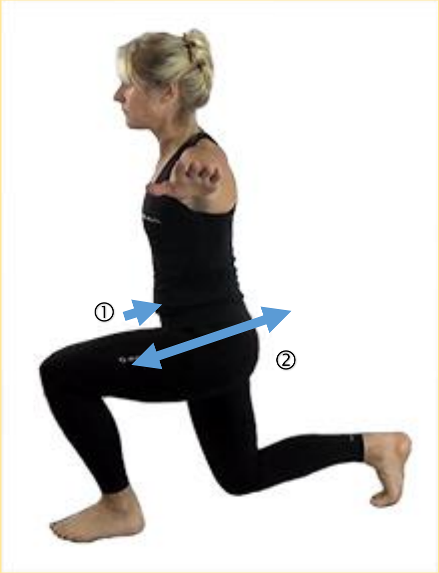

10C) Lunges with simultaneous upper body movement

Stand with your feet hip width apart and your arms up horizontal to your body.

① Start with ”core activation”.

② Maintain a straight back posture while you perform forward lunges by taking av step forward with your weight over that leg och then taking a step back again. Alternate which foot you step forward with. At the same time as you lung, try lifting upp your arms over your head or rotating your upper body from side to side when holding a stick.

Repetitions________________ each side

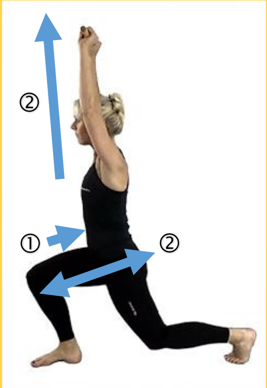

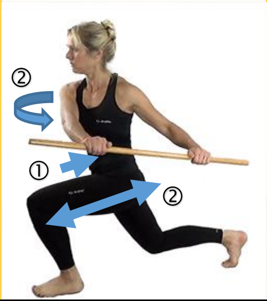

©Linköping University 01042017

**Training range of movement**		
**1A) Backward bending (elbow support)**<br><br>Lay on your stomarch and support yourself on your underarms/elbows. Bend your back backwards by pressing up from your underarms/elbows and return to the start position again.<br>Repetitions________________<br>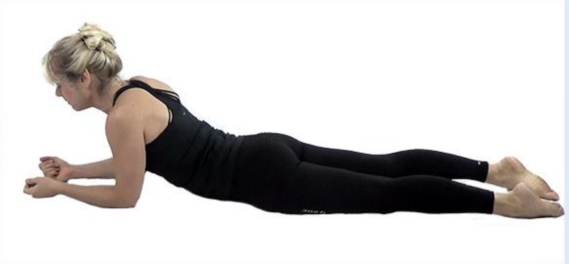	**1B) Backward bending (bent arms)**<br><br>Lay on your stomarch and support yourself with your hands. Bend your back backwards by pressing up from your hands but dont straighten your elbows and thereafter return to the start position again.<br>Repetitions________________<br>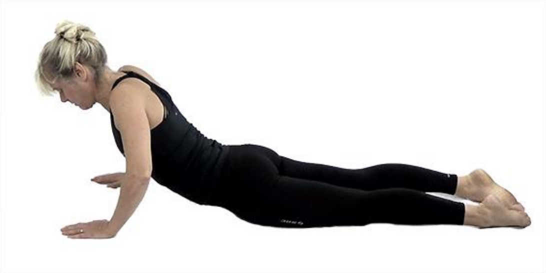	**1C) Backward bending (straight arms)**<br><br>Lay on your stomarch and support yourself with your hands. Bend your back backwards by pressing up from your hands and straightening your elbows and thereafter return to the start position again.<br>Repetitions________________<br>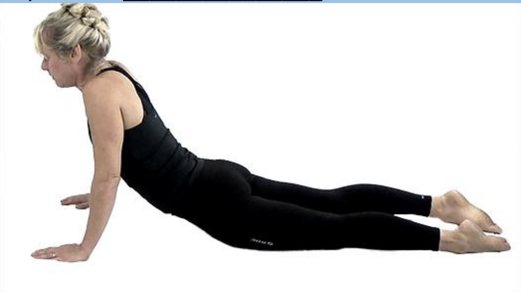
**2A) Foward bending while laying on your back**<br>Lay on your back and bring your knees up to your stomach, then return to the start position.<br><br>Repetitions________________<br>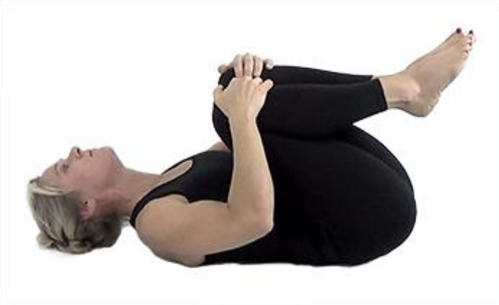	**2B) Forward bending on hands and knees**<br>Position yourself on your hands and knees with your back straight. Bend your back forward pressing your lower back upwards while bending your hips and knees so that your knees are in contact with your chest. Return to the starting position.<br><br>Repetitions________________<br>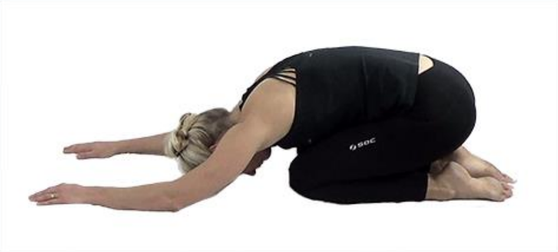	**2C) Forward bending in sitting or standing**<br>Stand/sit with your back straight. Starting bending forwards nd bringing your hands down towards the floor. Try to even bend your lower back. Return to your starting position.<br><br>Repetitions________________<br>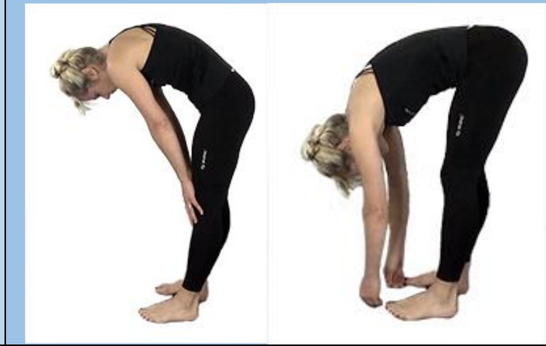

©Linköping University 01042017

**3A) Back rotation (lower back)**<br>Lay on your back and bring your knees down towards the floor on onside and then over to the other side.<br><br>Repetitions________________ each side<br>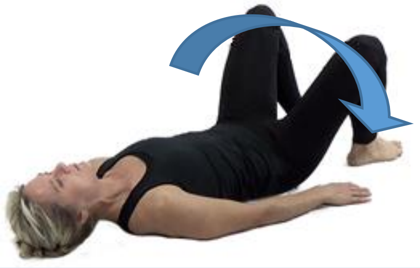	**3B) Back rotation (lower back and thoracic)**<br>Lay on your back and bring your knees down towards the floor on one side while simultaneously reaching out with your opposite arm upwards and sidewards. Change sides by bringing your knees over to the other side and reach out with your opposite arm upwards and sidewards.<br><br>Repetitions________________ each side<br>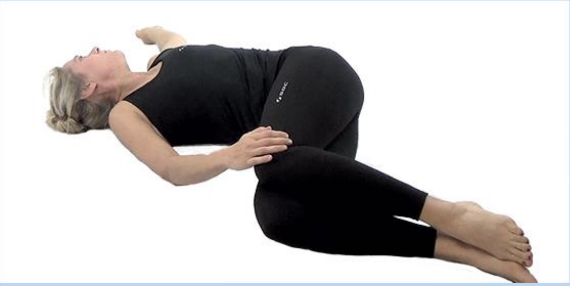	**3C) Back roation (full range)**<br>Lay on your back and bring your left knee down towards the floor on your left side while simultaneously reaching out with your left arm upwards and sidewards. Change sides by bringing your knee over to the other side and reach out with your opposite arm upwards and sidewards.<br><br>Repetitions________________ each side<br>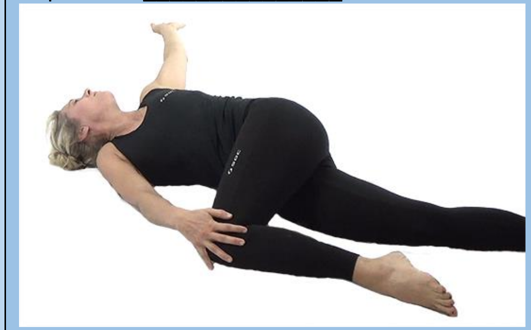
**Before and after exercise, stretching exercises help your muscles. Each stretch can be done several times, with <30 second holds. Here are suggestions for stretching.**	**Stretching of your buttock muscles**<br>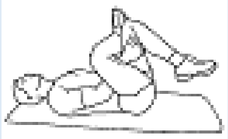	**Stretching of your hip muscles**<br>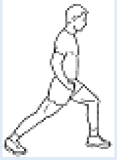
**Stretching of your thigh muscles**<br>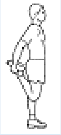	**Stretching of the back of your thighs**<br>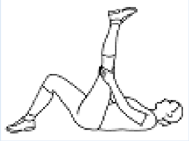	**Stetching of the inside of your thighs/groin**<br>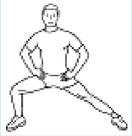

©Linköping University 01042017

General training - getting in shape

Training form

Regular physical exercise as a part of everyday life is important for maintaining good health and fitness. For this, we recommend following a training program prescribed by your physiotherapist. Your training can consist of, for example: walks, nordic walking, cycling, jogging, swimming, dancing, gym. Choose which training form is best for you. You can work out alone or with others in a group. The most important thing is that you feel that you take the time for physical activity in your everyday life.

Training intensity

Training intensity can be regulated through a so-called "pacing model". This means that you slowly and gradually increase your training intensity without overloading. You "pace" yourself in a controlled way to reach your goals. You can monitor your level of exertion by using a scale of 6-20 where the scale is based on your approximate pulse when you multiply by 10.

You should preferably training with a level of exertion between

11 (fairly light) and 14 (somewhat hard).

You should start exercising at about 20% less duration than you are capacble of. If you feel that the exercise feels very easy (at level 9 or below), you can increase your exercise duration slightly so that you feel at least a farily light exertion level (level 11).

When you experience your exercise exertion is on average under a "somewhat hard" lavel (below 14), you can increase your exercise by 20% after 2 weeks. If you are on level 15 or more, you can continue with the same training for an additional 2 weeks.

When your training duration lasts 30 minutes, you can increase the load by increasing the intensity to 15/16 (Hard - you can not speak on at this intensity) in 10 minute intervals. Then you can increase the number of minutes on this intensity (15/16) every second week.

If you have a bad day, you should work out half of what you planned. In this way you can increase your exercise gradually, without risking doing too much.

Training Contract:

I will perform …………………………….. as my training form
I will train 3 times/week
I will begin with …………. minutes
I will increase my training intensity with 20 % every second week until reach my goal capacity.

©Linköping University 01042017

Rating of Perceived Exertion
Borg RPE Scale

6		How you feel when lying in bed or sitting in a chair relaxed. Little or no effort.
7	Very, very light	
8		
9	Very light	
10		
11	Fairly light	
12		Target range: How you should feel with exercise or activity.
13	Somewhat hard	
14		
15	Hard	
16		
17	Very hard	How you felt with the hardest work you have ever done.
18		
19	Very, very hard	
20	Maximum exertion	Don’t work this hard!

©Linköping University 01042017

Training diary

Name:

Your physiotherapist will fill in which exercises you should train. You can cross off when you have performed the exercises.

Week	Day	BetterBack☺ Part 1			BetterBack☺ Part 2										BetterBack☺ Range of movement			General training
		**1**	**2**	**3**	**1**	**2**	**3**	**4**	**5**	**6**	**7**	**8**	**9**	**10**	**1**	**2**	**3**	**Borgskalan**
	Mon																	
	Tue																	
	Wed																	
	Thu																	
	Fri																	
	Sat																	
	Sun																	
	Mon																	
	Tue																	
	Wed																	
	Thu																	
	Fri																	
	Sat																	
	Sun																	
	Mon																	
	Tue																	
	Wed																	
	Thu																	
	Fri																	
	Sat																	
	Sun																	
	Mon																	
	Tue																	
	Wed																	
	Thu																	
	Fri																	
	Sat																	
	Sun																	
	Mon																	
	Tue																	
	Wed																	
	Thu																	
	Fri																	
	Sat																	
	Sun																	
	Mon																	
	Tue																	
	Wed																	
	Thu																	
	Fri																	
	Sat																	
	Sun																	

©Linköping University 01042017
